# Resting-State EEG Microstate Dynamics as Neurophysiological Biomarkers Across the Alzheimer’s Disease Continuum: A Systematic Review

**DOI:** 10.3390/diagnostics16183019

**Published:** 2026-09-17

**Authors:** Chanda Simfukwe, Seong Soo A. An, Young Chul Youn

**Affiliations:** 1 Department of Bionano Technology, Gachon University, 1342 Seongnam-daero, Seongnam-si 13120, Republic of Korea; chandaelizabeth94@gmail.com; 2 Department of Neurology, College of Medicine, Chung-Ang University, Seoul 06974, Republic of Korea; 3 Department of Medical Informatics, College of Medicine, Chung-Ang University, Seoul 06974, Republic of Korea

**Keywords:** Alzheimer’s disease, mild cognitive impairment, subjective cognitive decline, EEG microstates, quantitative EEG, neurophysiological biomarkers, AT(N) framework, systematic review

## Abstract

**Background/Objectives:** Alzheimer’s disease (AD) is preceded by clinically defined syndromes of subjective cognitive decline (SCD) and mild cognitive impairment (MCI), which are etiologically heterogeneous and belong to the AD continuum only when amyloid and tau pathology is biologically confirmed. Electroencephalography (EEG) microstates, brief periods of quasi-stable large-scale neural synchrony, have emerged as candidate markers of resting-state network disruption. This systematic review evaluated EEG microstate differences across clinically defined SCD, MCI, and AD dementia samples and the extent to which these have been related to amyloid/tau/neurodegeneration (AT(N)) biomarkers. **Methods:** PubMed/MEDLINE and EMBASE were searched (January 1990 to June 2026) on 30 June 2026 following preferred reporting items for systematic reviews and meta-analyses (PRISMA) 2020 guidelines, with an expanded search of Scopus, IEEE Xplore, Web of Science, PROSPERO, ClinicalTrials.gov, and WHO International Clinical Trials Registry Platform (searched 5 August 2026). Twenty-six primary studies met the eligibility criteria and were synthesized narratively. Two recently published meta-analyses identified by the same search were not counted among the included studies; they were appraised using AMSTAR 2 and used only to benchmark the primary-study synthesis, avoiding double counting of overlapping datasets. Methodological quality was appraised using the Newcastle–Ottawa Scale adapted for cross-sectional studies. **Results:** A graded pattern of microstate differences was identified across diagnostic groups. Microstate A duration and coverage were significantly increased at both the MCI and AD dementia stages. Microstate D duration, occurrence, and coverage were reduced at the MCI stage in the pooled contextual estimates, although only two independent primary samples contributed a microstate D contrast at this stage, whereas only microstate D occurrence was significantly reduced at the AD dementia stage. Microstate C occurrence was significantly reduced at the AD dementia stage in the pooled contextual estimate, but no individual primary sample showed a significant reduction and two of six reported an increase, so the pooled and primary-study evidence diverge for this parameter. Reductions in microstate C at the SCD stage were reported by a single primary study and were associated with cerebrospinal fluid amyloid-β pathology. The direction of effect was not uniform across primary studies at any stage, and the number of independent samples contributing to each comparison ranged from none to six. Methodological heterogeneity limited direct cross-study comparability. **Conclusions:** Resting-state EEG microstate parameters show graded group-level differences across clinically defined SCD, MCI, and AD dementia samples. Because the evidence base is predominantly cross-sectional and only four of the 26 studies confirmed underlying AD pathology biologically, these findings should be interpreted as preliminary group-level neurophysiological correlates rather than as established diagnostic biomarkers or as evidence of early-detection or clinical-staging utility. Longitudinal cohorts and diagnostic-accuracy studies in AT(N)-stratified samples are required before any clinical application can be considered.

## 1. Introduction

Alzheimer’s disease (AD) and related dementias constitute one of the most pressing public health crises of the twenty-first century. In 2019, an estimated 57 million people were living with dementia worldwide, a figure projected to rise to approximately 152 million by 2050 as global populations age [1]. AD accounts for 60–70% of all dementia cases, and its global economic cost already exceeds one trillion USD annually [2]. The 2020 Lancet Commission on dementia prevention, intervention, and care estimated that up to 40% of dementia cases may be attributable to 12 modifiable risk factors, underscoring the critical importance of identifying individuals at risk before clinical symptoms emerge [3]. In the United States alone, approximately 6.9 million people aged 65 and older currently live with AD, at an estimated annual care cost of 360 billion USD [4]. These figures collectively underscore the urgent need for scalable, accurate, and cost-effective biomarkers capable of detecting AD-related neurophysiological changes at the earliest possible stages.

The pathological process underlying AD begins decades before the onset of detectable cognitive symptoms [5,6]. The National Institute on Aging Alzheimer’s Association (NIA-AA) Research Framework operationalized a biological definition of AD based on an AT(N) classification system encompassing amyloid-β deposition (A), pathological tau (T), and neurodegeneration or neuronal injury (N) [4]. This framework reframes AD as a biological continuum rather than a purely clinical syndrome, enabling staging of disease pathology independent of symptom expression. Amyloid-β accumulation is understood to precede tau propagation and neurodegeneration by 15 to 20 years, following a temporal biomarker cascade conceptualized in the dynamic biomarker model of Jack and colleagues [5]. Advanced plasma biomarkers have rapidly updated the AT(N) framework in recent years; in particular, phosphorylated tau-217 (p-tau217) demonstrates an area under the receiver operating characteristic curve of approximately 0.96 for distinguishing AD from other neurodegenerative conditions [7]. Despite these advances, plasma p-tau217 and related markers require dedicated laboratory infrastructure, and cerebrospinal fluid (CSF) collection and amyloid positron-emission tomography (PET) imaging remain invasive or costly, limiting their widespread adoption and motivating the search for complementary neurophysiological biomarkers [8].

### 1.1. The Alzheimer’s Disease Continuum and Early Biomarker Needs

Clinically, AD unfolds across a staged continuum encompassing cognitively unimpaired individuals with underlying pathology, a preclinical symptomatic phase known as subjective cognitive decline (SCD), a prodromal symptomatic phase termed mild cognitive impairment (MCI), and finally dementia. This continuum was formalized through NIA-AA diagnostic criteria establishing standardized definitions for preclinical AD, MCI due to AD, and dementia due to AD [9,10,11]. Early identification of individuals at the SCD and MCI stages is considered essential for any strategy aimed at secondary prevention or disease modification [3,9].

The SCD occupies a critical position within the AD continuum. It is defined as a self-experienced persistent decline in cognitive capacity relative to a previous level, occurring in the context of normal performance on standardized neuropsychological tests [11]. A meta-analysis encompassing 28 cohort studies involving 29,723 individuals demonstrated that people with SCD carry approximately twice the risk of developing dementia compared to those without subjective complaints (RR = 2.07) [12]. The NIA-AA preclinical staging framework delineates three sequential phases: asymptomatic amyloidosis, synaptic dysfunction, and subtle cognitive change, suggesting that neurophysiological abnormalities may be detectable even prior to overt cognitive complaints [9]. Despite this evidence, SCD remains one of the most underrepresented stages in neurophysiological biomarker research.

MCI, formalized as a clinical diagnostic entity by Petersen [13], describes a state of cognitive decline exceeding what is expected for age and education, but insufficient to impair daily functional activities. Preserved functional independence is the defining criterion distinguishing MCI from frank dementia, as clarified by the Stockholm MCI consensus [14]. Amnestic MCI in particular carries a conversion rate to AD dementia of approximately 10–15% per year [15]. Population-based estimates suggest that MCI affects approximately 15–20% of adults aged 65 and over, representing a substantial window for intervention if reliable progression biomarkers can be identified.

### 1.2. EEG Microstates as Candidate Neurophysiological Biomarkers

Electroencephalography (EEG) has attracted considerable interest as a neurophysiological biomarker in this context, owing to its millisecond temporal resolution, non-invasiveness, low cost, and scalability across diverse clinical settings [16]. An international expert panel endorsed resting-state EEG measures as viable outcomes for AD clinical trials, specifically citing their scalability and sensitivity to cortical network changes [17]. Quantitative EEG (qEEG) measures, including theta/alpha power ratios and global field synchronization, have demonstrated sensitivity to AD-associated neurophysiological changes [17,18]. Characteristic EEG slowing, manifested as increased theta power and reduced alpha and beta activity, has been documented as a reproducible marker of cortical dysfunction in AD [19].

A particularly promising and methodologically distinct class of EEG-derived measures within this biomarker landscape is EEG microstates, first formalized by Lehmann and colleagues in 1987 as discrete, quasi-stable periods of scalp electric field topography lasting approximately 60–120 ms before transitioning abruptly to a new configuration [20]. These recurring topographic states, identifiable in continuous resting-state EEG recordings, represent the brain’s spontaneous organization into transient large-scale network configurations and constitute the fundamental temporal building blocks of cortical dynamics at the millisecond scale [21,22]. Four canonical classes, conventionally labelled A to D, are recovered consistently across large participant samples and are summarized by a small set of temporal parameters [21,22,23,24,25,26]. The segmentation algorithms used to derive them, the parameters extracted from them, and the methodological debate surrounding both bear directly on how the evidence was appraised in this review and are therefore set out where they are applied: in the eligibility and data-extraction criteria (Section 2.2 and Section 2.5) and in the appraisal of methodological heterogeneity and standardization (Section 4.4).

The biological significance of EEG microstates is grounded in their established correspondence with hemodynamic resting-state networks identified via functional magnetic resonance imaging (fMRI) [27]. Concurrent EEG-fMRI work demonstrated that microstate A co-activates auditory and language networks, microstate B co-activates visual networks, microstate C co-activates regions associated with the default mode network (DMN) [27,28] and salience network, and microstate D co-activates frontal and parietal regions of the attentional control network [26]. A systematic review confirmed that microstate C is functionally associated with DMN-dependent processes including mind-wandering, autobiographical memory, and self-referential cognition, while microstate D is linked to cognitive control and attentional orienting [28].

### 1.3. Rationale and Aim of the Review

Converging evidence suggests that EEG microstate dynamics differ across clinically defined SCD, MCI, and AD dementia groups, raising the possibility that they may provide complementary neurophysiological information across these syndromes. The most comprehensive meta-analysis to date synthesized data from 30 studies and reported that microstate A duration is significantly prolonged in AD and that microstate D occurrence is reduced in MCI [29,30]. Substantial inter-study heterogeneity (I^2^ > 60%) attributed to methodological inconsistencies was also identified [30]. An independent meta-analysis encompassing 12 studies and 1347 participants similarly identified increased microstate A duration and reduced microstate D activity in MCI and AD, alongside reductions in microstate C and D coverage in established AD [30,31].

Critically, Lassi and colleagues provided the first direct evidence that microstate degradation, particularly involving microstate C, is already detectable at the SCD stage and correlates with CSF amyloid-β42 levels, outperforming conventional spectral EEG measures in discriminating SCD from healthy controls [31,32]. The only published longitudinal microstate study in MCI demonstrated that microstate D dynamics were the strongest differentiator between progressive and stable MCI over a three-year follow-up [32]. Furthermore, CSF amyloid-β1-42 concentrations have been shown to correlate specifically with microstate B occurrence and microstate C duration in AD, providing a preliminary molecular-neurophysiological link [33]. A large-scale cross-sectional study involving 945 participants spanning SCD, MCI, and AD identified a systematic gradient of microstate A-to-B shift across the clinical continuum [34,35]; yet no systematic review has synthesized these findings within a unified stage-by-stage framework.

Despite this accumulating evidence, significant gaps remain. No published systematic review has examined EEG microstate alterations comprehensively and comparatively across all stages of the AD continuum from healthy ageing through SCD, MCI, and AD dementia within a single integrated analysis. The most recent meta-analyses have focused primarily on MCI and AD, with SCD substantially underrepresented [29]. Methodological heterogeneity, including variability in electrode density, reference scheme, clustering algorithm, and the number of extracted microstate classes, further limits cross-study comparability [24]. Importantly, no review has examined whether EEG microstate parameters map onto the AT(N) biomarker framework [5], despite the theoretical relevance of DMN-linked microstate C to amyloid-associated network disruption and of frontoparietal microstate D to tau-associated attentional and executive decline. A recent expert review of quantitative EEG biomarkers in AD and MCI explicitly identified the absence of a review integrating microstate staging with the AT(N) framework as a key unresolved gap in the field [34,35,36].

**Figure 1:**
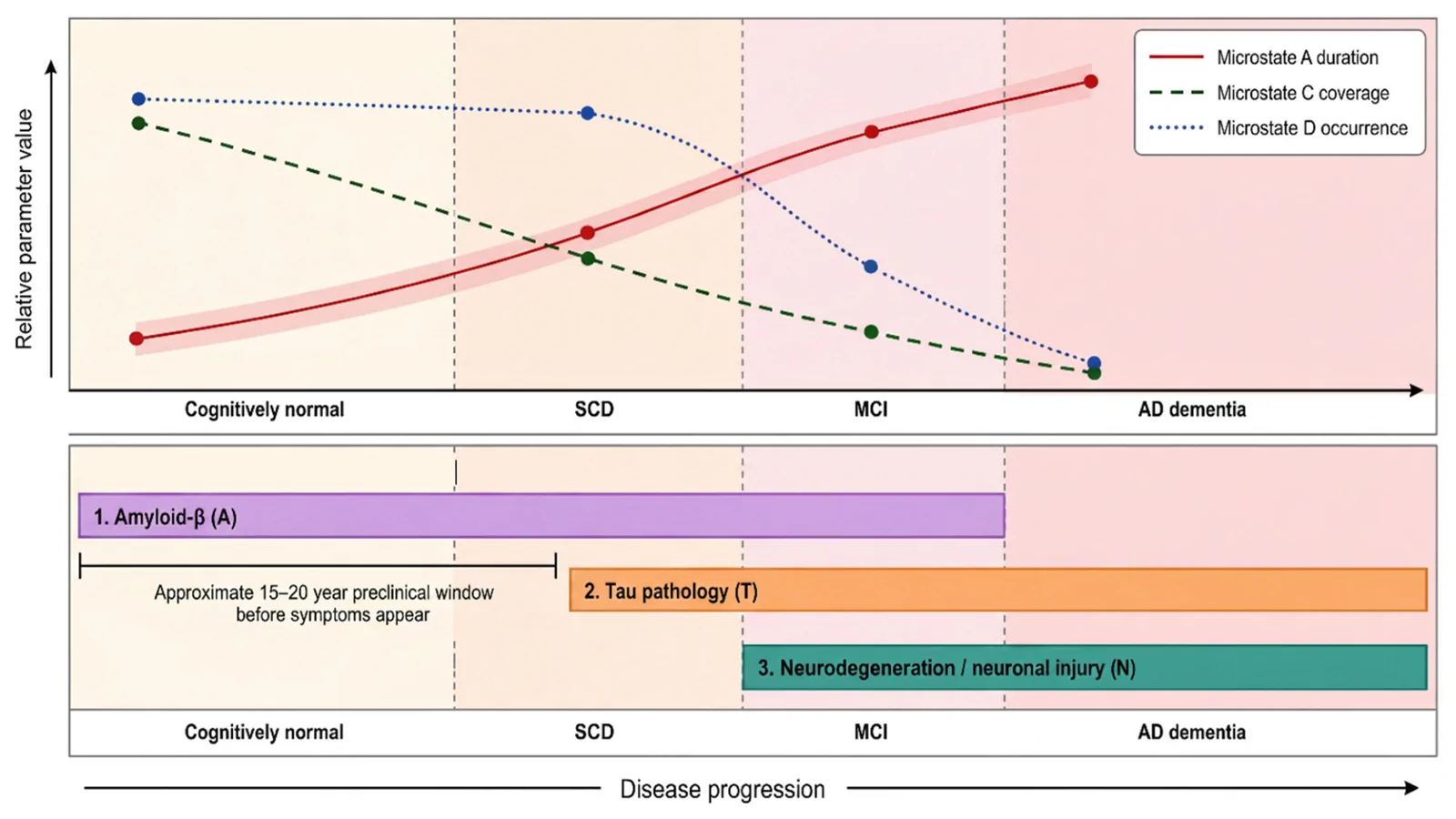
Conceptual schematic illustrating the hypothesized staging of EEG microstate changes across the AD continuum. The trajectories shown are hypothetical and illustrative; they represent a conceptual model motivating this review rather than an empirically established staging pattern, and no included study measured these parameters longitudinally within individuals. **Upper** panel: Hypothetical trajectories of three EEG microstate parameters across four clinical stages. Microstate A duration (red solid line) increases progressively from the cognitively normal stage through to AD dementia [31,33]; microstate C coverage (green dashed line) declines from the SCD stage onward [32,35]; microstate D occurrence (blue dotted line) declines sharply from MCI onward [31]. The shaded band indicates uncertainty around the microstate A trajectory; all trajectories are conceptual and illustrative of synthesized evidence and are not derived from a single empirical dataset. **Lower** panel: AT(N) biomarker cascade showing the established temporal ordering of amyloid-β deposition (A), tau pathology (T), and neurodegeneration/neuronal injury (N) [4], with an approximate 15–20-year preclinical window preceding symptom onset. Abbreviations: EEG, electroencephalography; SCD, subjective cognitive decline; MCI, mild cognitive impairment; AD, Alzheimer’s disease; AT(N), amyloid/tau/neurodegeneration; CN, cognitively normal; CSF, cerebrospinal fluid; DMN, default mode network.

The present systematic review was conducted in accordance with preferred reporting items for systematic reviews and meta-analyses (PRISMA) 2020 guidelines [37] to address these gaps. The primary objective is to systematically identify, appraise, and synthesize evidence on EEG microstate dynamics as staged neurophysiological biomarkers across the full AD continuum, from subjective cognitive decline to established dementia, including their associations with AT(N) biomarkers, cognitive measures, and neuroimaging findings. Secondary objectives include evaluating methodological consistency across studies and identifying priorities for future longitudinal and translational research. The hypothesized stage-by-stage pattern of microstate change, considered in relation to the AT(N) biomarker cascade, is illustrated schematically in Figure 1; this schematic represents the conceptual model motivating the review and is not a synthesis result.

## 2. Materials and Methods

### 2.1. Protocol Registration and Reporting Standards

This study is a systematic review of primary studies with a structured narrative synthesis; it is not an overview of reviews. The two systematic reviews with meta-analysis identified by the search are handled as a separate contextual reference tier rather than as included evidence, and the resulting two-tier structure is specified in Section 2.7. This systematic review was not prospectively registered in a public registry. The eligibility criteria, search strategy, and planned approach to data synthesis were finalized in accordance with PRISMA 2020 reporting standards [37] prior to the commencement of full-text screening and data extraction. This a priori protocol was prepared for internal use only; it was neither registered in a public registry nor published and is therefore not publicly accessible. After completion of the initial searches, the information-source strategy was amended to include Scopus, IEEE Xplore, Web of Science Core Collection, PROSPERO, ClinicalTrials.gov, and WHO ICTRP. These additional searches were conducted on 5 August 2026. Two amendments were made to the a priori eligibility criteria after the initial searches had been completed, and both are reported here for transparency. First, the list of acceptable diagnostic frameworks was broadened beyond NIA-AA, DSM-5, and ICD to include other internationally validated criteria applied in the source studies (NINCDS-ADRDA, DSM-IV, IWG-II, SIDAM, and the SCD-I research criteria); this was necessary because the protocol list, drawn from contemporary guidelines, would otherwise have excluded studies published before the NIA-AA criteria were issued in 2011. Thirteen of the 26 included studies rest wholly or partly on these frameworks (Supplementary Table S1); applying the original list would have removed the entire pre-2011 literature. Second, the comparator requirement was relaxed from an age- and sex-matched control group to an age-matched control group as the minimum, because sex matching was frequently not stated explicitly even where group sex distributions were reported; no study was included on the basis of this relaxation that would not also have met the original requirement had sex distribution been reported. No changes were made to the index-test definition, the outcome definitions, or the planned synthesis framework. Both amendments were applied uniformly to all records at the re-screening stage described in Section 3.1, so that every study was assessed against the same final criteria. To quantify the effect of these amendments on study selection, a post hoc sensitivity analysis was conducted in which the original a priori list of diagnostic frameworks was reapplied to all 26 included studies; the outcome is reported in Section 3.1 and the study-by-study classification in Supplementary Table S7.

### 2.2. Eligibility Criteria

Study eligibility was defined a priori in accordance with the PICO framework (Population, Index test, Comparator, Outcome) [38]. Regarding the study population, eligible studies were required to include human adults aged 50 years or older with a clinical or research diagnosis of SCD, MCI, or AD dementia, established in accordance with NIA-AA 2011 or 2018 diagnostic criteria [4,9,10], the Diagnostic and Statistical Manual of Mental Disorders Fifth Edition (DSM-5), or the International Classification of Diseases (ICD-10 or ICD-11), or another internationally validated diagnostic framework applied in the source study (including NINCDS-ADRDA, DSM-IV, IWG-II, SIDAM, or the SCD-I research criteria for subjective cognitive decline). This last provision is an amendment to the a priori criteria, reported in Section 2.1; it was applied uniformly to all records at re-screening. The NIA-AA criteria were further revised in 2024 [39]; because that revision post-dates the great majority of the included studies and was applied by none of them (Section 3.2.1), it was not added to the list of acceptable frameworks. With respect to the index test, eligible studies were required to have employed resting-state EEG microstate analysis using any validated segmentation algorithm, including but not limited to modified K-means clustering [22], atomize and agglomerate hierarchical clustering (AAHC) [40], or independent component analysis (ICA) [21,22]. The required comparator was an age- and sex-matched neurologically healthy control group, against which group differences in microstate parameters could be meaningfully evaluated. Where sex matching was not explicitly reported, an age-matched neurologically healthy control group was accepted as the minimum comparator requirement. This relaxation is likewise an amendment to the a priori criteria (Section 2.1). In terms of outcomes, studies were required to report at least one quantitative standardized microstate temporal parameter, including mean duration (ms), occurrence rate (events/s), global explained variance or coverage (%), transition probability, or microstate sequence syntax entropy [21,29].

Studies were excluded from consideration if they met any of the following criteria: conference abstracts, preprints, non-peer-reviewed publications, book chapters, or editorials; non-human, animal model, or in vitro studies; studies failing to report at least one extractable quantitative microstate parameter; studies of mixed or non-AD dementia syndromes without a dedicated AD-spectrum subgroup analysis; studies lacking an age-matched healthy control comparator group; studies employing exclusively task-based or sleep EEG paradigms without a resting-state recording epoch; and studies published prior to January 1990, the start date of the systematic search. The foundational EEG microstate conceptual framework was established by Lehmann and colleagues in 1987 [20] and is cited separately as a methodological background source.

### 2.3. Information Sources and Search Strategy

Systematic literature searches were initially conducted in PubMed/MEDLINE and EMBASE for studies published between 1 January 1990 and 30 June 2026. The final searches of these two databases were executed on 30 June 2026. Additional searches were conducted on 5 August 2026 in three bibliographic databases, Scopus, IEEE Xplore, and Web of Science Core Collection, and three registration sources: PROSPERO, ClinicalTrials.gov, and the World Health Organization International Clinical Trials Registry Platform (WHO ICTRP). The registries were searched to identify ongoing, prospectively registered, or unpublished studies and thereby reduce the risk of retrieval and reporting bias. The final search of both databases was executed on 30 June 2026. A human-species filter and a publication-date limit of 1 January 1990 to 30 June 2026 were applied in both databases. The following core search string was applied across all platforms and subsequently adapted using database-specific Boolean operators, controlled vocabulary comprising Medical Subject Headings (MeSH) terms for PubMed/MEDLINE and Emtree terms for EMBASE, and field-tag syntax:

(“EEG” OR “electroencephalograph*” OR “electroencephalogram”) AND (“microstate*”) AND (“Alzheimer*” OR “dementia” OR “mild cognitive impairment” OR “MCI” OR “subjective cognitive decline” OR “SCD” OR “cognitive impairment”).

The complete, database-specific search strategies for PubMed/MEDLINE and EMBASE, including the full Boolean structure, all field tags, the MeSH and Emtree controlled-vocabulary terms, the applied species and date filters, and the exact date of the final search, are reported verbatim in Supplementary Table S3, so that the searches can be reproduced exactly. The source-specific strategies, execution dates, and record yields for Scopus, IEEE Xplore, Web of Science Core Collection, and the three registration sources are reported in Supplementary Table S3, Sections D–I. No language restriction was applied during the initial database search phase to minimize retrieval bias; however, at the full-text eligibility assessment stage, only English-language articles were retained, as non-English full texts could not be evaluated for methodological rigor with sufficient reliability. To supplement electronic database searches and further minimize the risk of publication bias, the reference lists of all included studies and of relevant systematic reviews identified during screening were hand-searched for additional eligible records. This backward citation search was performed manually by one reviewer and cross-checked by a second; any additional records identified were subjected to the same two-stage screening process as database-retrieved records.

### 2.4. Study Screening and Selection Procedures

All 292 records retrieved from the two database searches were imported into Rayyan (Qatar Computing Research Institute, Doha, Qatar) [41]; duplicate records were removed using Rayyan’s automated deduplication function with manual verification, leaving 191 unique records for blinded, independent screening. Study selection was conducted in two sequential, prespecified stages. In the first stage, title and abstract screening, all deduplicated records were independently assessed by two reviewers against the prespecified eligibility criteria, with each reviewer blinded to the decisions of the other. In the second stage, full-text eligibility assessment, all records passing Stage 1 were retrieved in full text and independently evaluated by both reviewers against the complete set of inclusion and exclusion criteria. Discrepancies arising at either stage were resolved through structured discussion between the two reviewers; cases in which consensus could not be reached were referred to a third independent reviewer for adjudication.

**Figure 2:**
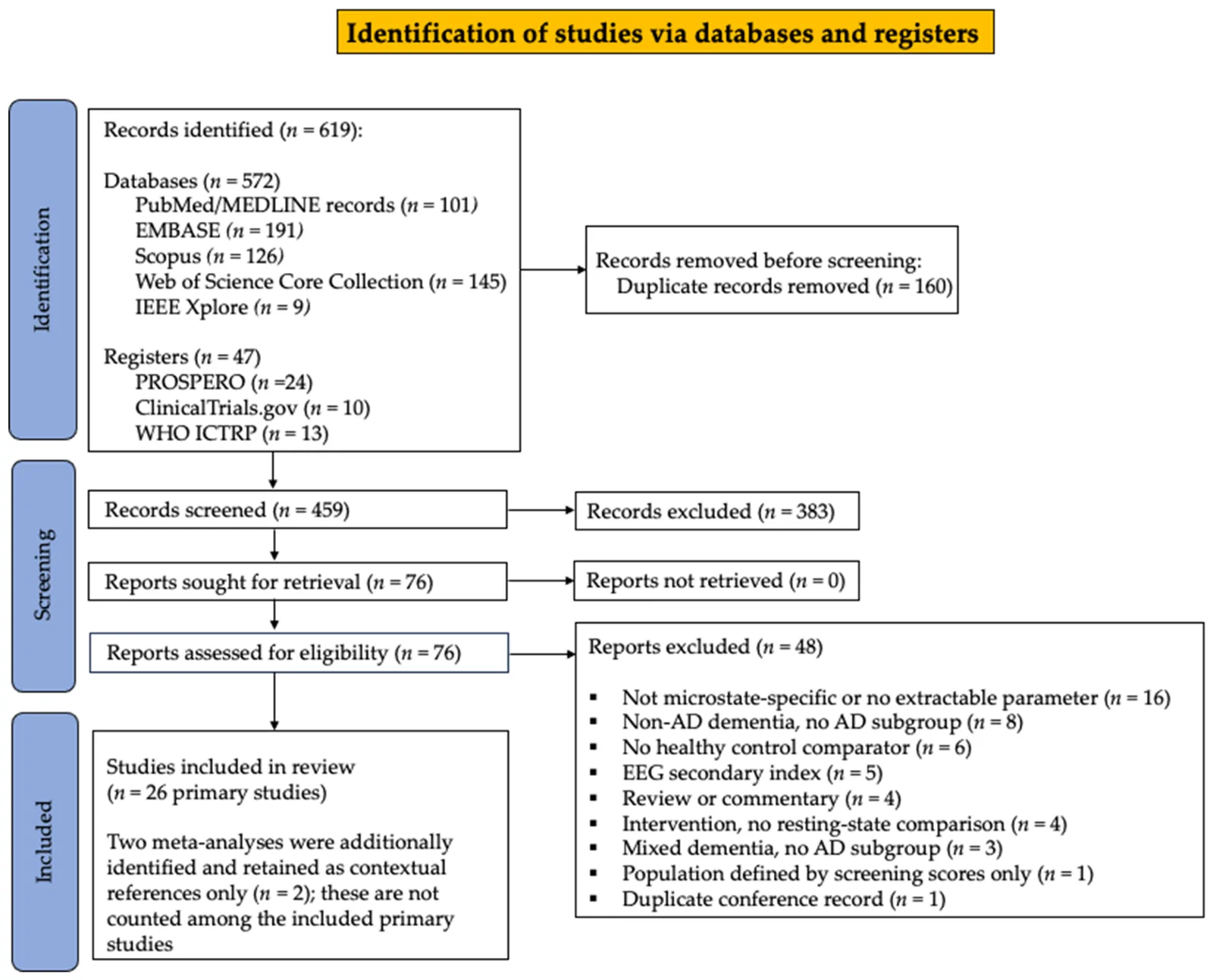
PRISMA 2020 flow diagram of study identification, screening, eligibility assessment, and inclusion. Counts combine the original searches of PubMed/MEDLINE and EMBASE, executed on 30 June 2026, with the additional searches of Scopus, IEEE Xplore, Web of Science Core Collection, PROSPERO, ClinicalTrials.gov, and WHO ICTRP, conducted on 5 August 2026, as described in Section 2.3. Reports excluded at the eligibility stage include three records excluded on re-screening against the a priori PICO criteria (Section 3.1). The two meta-analyses [30,31] identified by the search are retained as a separate contextual reference layer and are not counted among the 26 included primary studies. Full screening and exclusion details are reported in Section 3.1. Abbreviations: AD, Alzheimer’s disease; EEG, electroencephalography; *n*, number.

Inter-rater reliability was quantified using Cohen’s kappa (κ), calculated separately for the title and abstract screening stage and the full-text assessment stage, and interpreted using established benchmarks: κ = 0.41–0.60, moderate agreement; κ = 0.61–0.80, substantial agreement; κ = 0.81–1.00, almost-perfect agreement [42]. The complete study selection process, including the number of records identified, screened, excluded with reasons, and ultimately included, is presented in a PRISMA 2020-compliant flow diagram (Figure 2) [37].

### 2.5. Data Extraction

A standardized data extraction form was developed prior to full-scale extraction and piloted independently by both reviewers on three randomly selected included studies to confirm its comprehensiveness and inter-rater consistency before implementation across the full study set. Both reviewers subsequently extracted data from all included studies independently, and all discrepancies in extracted values were resolved by consensus and cross-verified against the source documents.

Variables were extracted from each included study under four headings. Study and participant characteristics comprised first author, year of publication, country of origin, study design, total and per-group sample size, mean age, sex distribution, years of formal education, and performance on cognitive assessment instruments per group (Mini-Mental State Examination, MMSE; Montreal Cognitive Assessment, MoCA; Clinical Dementia Rating, CDR), together with the diagnostic criteria used to define SCD, MCI, or AD (e.g., NIA-AA 2011/2018 [4,9,10], DSM-5, CDR staging, MMSE cut-off scores). Methodological variables comprised EEG acquisition parameters (recording system and manufacturer, number of electrodes, reference scheme, sampling rate, total artefact-free epoch length, and artefact rejection method) and microstate analysis parameters (segmentation algorithm [22,40], number of microstate classes, polarity handling, and minimum duration threshold). Outcome variables comprised duration (ms), occurrence (events/s), coverage (%), transition probability matrices, and sequence syntax entropy where reported [21,29], together with the direction and magnitude of group differences per microstate class, the statistical test applied, and effect size indices (Cohen’s d, partial η^2^, or equivalent) where available. Biomarker variables comprised reported associations between microstate parameters and AT(N) framework markers [4], including cerebrospinal fluid amyloid-β42/40 ratios, phosphorylated tau, amyloid or tau PET standardized uptake value ratios, MRI-derived volumetric indices, and neuropsychological composite scores.

### 2.6. Risk of Bias and Methodological Quality Assessment

The methodological quality of each included study was evaluated independently by both reviewers using the Newcastle–Ottawa Scale adapted for cross-sectional studies (NOS-CS) [43], selected given that the predominant anticipated study design across the literature is cross-sectional or case-control in nature. The NOS-CS was chosen because it is the most widely validated and transparent instrument for appraising non-randomized observational studies and, unlike a single global score, supports domain-level scoring across selection, comparability, and outcome, allowing specific methodological strengths and weaknesses to be identified. The NOS-CS appraises three domains: sample selection (maximum 5 stars), comparability of groups based on the study design or analysis (maximum 2 stars), and assessment of exposure and outcome (maximum 3 stars), yielding a maximum total score of 10 stars. Studies accumulating 7–10 stars were classified as high quality, those with 4–6 stars as moderate quality, and those with 0–3 stars as low quality. These bands, and the practice of summing stars across domains, are conventions adopted for descriptive purposes and are not externally validated: the Newcastle–Ottawa Scale has no established cut-points, and a summed score weights each item equally despite their unequal bearing on risk of bias. Domain-level judgements (Supplementary Table S2) are therefore the primary form in which methodological quality is reported; the summary score and bands are presented only as a descriptive overview and were not used to weight, rank, or exclude studies in the synthesis. To avoid rewarding incompletely reported studies, a star was awarded only where the relevant information was explicitly reported. Within the selection domain, the sample-size item required the number of participants per diagnostic group to be reported and to be adequate for stable group-level microstate estimation (a prespecified minimum grounded in prior microstate reliability work), and the diagnostic-criteria item required an explicitly named, validated diagnostic framework; studies that did not report per-group sample sizes, diagnostic criteria, or their analytical pipeline were not awarded the corresponding stars.

The protocol provided that, should any prospective longitudinal or cohort study be identified, the standard Newcastle–Ottawa Scale for cohort studies would be applied in place of the cross-sectional adaptation [43]. In the event that no included study was of prospective cohort design, the single study incorporating longitudinal follow-up Tait et al. (2020) [44] was appraised on its cross-sectional group comparisons, which are the analyses contributing to this synthesis. All 26 included studies were therefore appraised with a single instrument, and no scores derived from different Newcastle–Ottawa variants were combined in the summary statistics reported in Section 3.2.3. Risk-of-bias findings are reported narratively within the relevant results subsections and presented at the domain level (selection, comparability, and outcome) in Supplementary Table S2 rather than as overall star scores alone; quality scores were used to inform narrative statements about confidence in the evidence in Section 4; no formal certainty-of-evidence framework (e.g., GRADE) was applied, and no quantitative method for assessing risk of bias due to missing results (e.g., funnel-plot asymmetry or Egger’s test) was prespecified, as neither is applicable to a synthesis that does not pool primary data. The two published meta-analyses identified by the search were not appraised with the NOS-CS; they were instead evaluated using AMSTAR 2, with domain-level confidence judgements reported in Supplementary Table S4, and are used only as contextual references rather than being pooled with the primary studies.

### 2.7. Data Synthesis and Statistical Analysis

Given the substantial methodological heterogeneity anticipated across eligible studies arising specifically from variation in EEG acquisition parameters, microstate segmentation algorithms, the number of microstate classes employed (typically four to seven), and diagnostic classification instruments, a de novo quantitative meta-analysis was prespecified as conditional and feasibility-dependent rather than as the default synthesis approach [37].

The feasibility threshold for conducting an independent pooled analysis was set at three or more studies reporting an identical microstate parameter for an identical diagnostic group comparison with sufficient extractable numerical data. Where this threshold was met, and data were sufficiently homogeneous, a random-effects meta-analysis would be performed using pooled standardized mean differences (SMDs) with 95% confidence intervals (CIs), weighted by inverse variance, with statistical heterogeneity quantified using the I^2^ statistic and Cochran’s Q test [37]. I^2^ values exceeding 50% were to be interpreted as indicative of substantial heterogeneity and values exceeding 75% as indicative of considerable heterogeneity [37].

In practice, the prespecified feasibility threshold was not met in a form that would permit a valid independent pooled analysis. Three specific reasons account for this, extending beyond methodological heterogeneity alone. First, for the principal temporal parameters (duration, occurrence, and coverage of microstates A–D) across the MCI-versus-control and AD-versus-control comparisons, aggregate-data random-effects estimates have already been published in two recent meta-analyses (Das et al. 2026 [30]; Piton et al. 2026 [31]); because these pool largely the same primary cohorts included here, an additional pooling would be of limited incremental value. We note explicitly that re-analyzing these primary cohorts would not itself constitute double counting: double counting arises only where the same data enter a single pooled estimate more than once, which is not the case when primary studies are pooled afresh. The constraint is therefore one of incremental value rather than of validity. To quantify rather than assume this overlap, the primary studies contributing to each meta-analysis were extracted from their reference lists and cross-tabulated against one another and against the present included set; this citation matrix, together with the corrected covered area (CCA) statistic, is reported in Supplementary Table S6. Of the 26 primary studies included here, 20 also contributed to the meta-analysis by Das et al. [30] and 10 to that by Piton et al. [31], yielding a CCA of 57.7%, which corresponds to very high overlap on the interpretive bands. Where the two meta-analyses report concordant estimates, that concordance is therefore described as consistency arising from shared source data rather than as independent replication. Second, microstate class labels are not interchangeable across studies unless the underlying template maps are re-sorted to a common reference, which cannot be performed post hoc from the summary statistics reported in primary articles; a parameter labelled for a given class in one study therefore does not necessarily correspond to the same scalp topography in another, precluding valid alignment of outcomes for pooling. Third, for outcomes not covered by the existing meta-analyses, notably transition probabilities, sequence and complexity entropy, the subjective-cognitive-decline-versus-control comparison, and direct MCI-versus-AD contrasts, fewer than three studies reported commensurable effect sizes together with the dispersion statistics (standard deviations or confidence intervals) required for inverse-variance weighting; several reported only effect directions, medians, or classification metrics, and parameters were expressed on non-commensurable scales (absolute versus ratio-normalized or z-scored values). A per-outcome account of the studies and statistics available, and the specific barrier to pooling for each outcome, is provided in Supplementary Table S5. Because several of the principal comparisons are supported by more than three primary studies, the analytical parameters bearing on whether those studies are commensurable are reported for every study in Supplementary Table S8: 18 of the 26 studies extracted a four-class solution, and after excluding reports whose quantities are not the canonical temporal parameters, are restricted to a single frequency band, or are ratio-normalized, PCA-transformed, or derived indices, nine studies remain that could enter a common pooled estimate in principle. Accordingly, the primary synthesis approach adopted was a structured narrative synthesis, following the Synthesis Without Meta-Analysis (SWiM) reporting guidance [45]. In the synthesis, each parameter-by-stage outcome is characterized by the number of independent participant samples contributing to it and the number of those samples reporting an increase, a decrease, or no significant difference relative to healthy controls. No composite grade is assigned; the constituent quantities are reported directly in Table 1 so that concordance of direction, the number of contributing samples, and the methodological quality of those samples can be weighed separately. To maintain a clear hierarchy of evidence and avoid double counting, this review adopts a two-tier evidence structure. The 26 primary studies constitute the included evidence base and were synthesized narratively. The two recently published meta-analyses identified by the search, Das et al. (2026) [30] and Piton et al. (2026) [31], were not treated as included studies, and their pooled samples were not combined with the primary evidence, because both meta-analyses pool many of the same primary studies included here; counting them as additional evidence alongside those same primary studies would double-count overlapping datasets and overstate convergence. They were instead retained as a separate contextual reference layer: each was formally appraised using AMSTAR 2 (Section 2.6; Supplementary Table S4), and their pooled effect estimates are reported only to benchmark the direction and magnitude of the primary-study synthesis, clearly distinguished from it throughout the results.

The narrative synthesis was organized along three prespecified dimensions: clinical stage along the AD continuum (SCD, MCI, and AD dementia); microstate class (A, B, C, D, and extended classes where applicable); and microstate parameter type (duration, occurrence, coverage, transition probability, and syntax entropy). Consistency of effect direction across studies was systematically evaluated and tabulated to facilitate cross-study comparison (Table 1). Because several publications analyze shared participant samples, a prespecified clustering rule was applied before any counting of studies. Publications were grouped into independent-sample clusters on the basis of the participant cohort analyzed rather than the publication unit, using the source dataset or recruiting cohort reported by the authors. Two such clusters were identified: the publicly available OpenNeuro cohort (ds004504), analyzed by Wan et al. (2024) [46], Yang et al. (2024) [47], Chang (2025) [48], and Hou et al. (2025) [49] and the companion reports of Dierks et al. (1997) [50] and Strik et al. (1997) [51], which describe a single German cohort. Lv et al. (2026) [52] draws on the shared BrainLat repository but is the only included study to do so and therefore forms a cluster of one. Within each cluster, the constituent publications contribute a single vote to the direction-of-effect tally for any given parameter-by-stage outcome, so that a shared cohort cannot be counted more than once; where publications within a cluster disagree in direction, the cluster is recorded as inconsistent rather than allowing the within-cluster majority to prevail. The same rule governs the study counts reported in Table 1, so that k refers to the number of independent samples rather than the number of publications. Cluster membership is identified in Supplementary Table S1, and the clustering does not alter the number of included studies reported in Section 3.1, which remains a count of publications. Where individual studies reported associations between microstate parameters and AT(N) biomarker indices, these findings were synthesized separately and are presented in Section 3.4.

## 3. Results

### 3.1. Study Selection

The electronic database searches of PubMed/MEDLINE and EMBASE identified 101 and 191 records, respectively, yielding 292 records in total prior to deduplication. Following removal of 101 duplicate records, 191 unique records proceeded to title and abstract screening. Of these, 129 were excluded at the title and abstract stage for the following reasons: studies not employing EEG microstate analysis as the primary index test (*n* = 52); populations outside the AD continuum (*n* = 28); absence of an age-matched healthy control comparator group (*n* = 19); conference abstracts, preprints, or editorials (*n* = 15); non-human or in vitro studies (*n* = 8); and absence of a quantitative microstate parameter (*n* = 7). Sixty-two full-text articles were assessed for eligibility, of which 35 were excluded for the following reasons: intervention or therapeutic study without an independent resting-state comparison against healthy controls (*n* = 4); non-AD dementia population without a dedicated AD-spectrum subgroup (*n* = 8); general EEG analysis without confirmed microstate-specific parameter reporting (*n* = 8); mixed dementia types without an AD-specific subgroup analysis (*n* = 3); review or commentary rather than original empirical study (*n* = 3); multimodal engineering or system paper in which EEG microstates were a secondary index (*n* = 5); study lacking an age-matched healthy control comparator group (*n* = 3) and duplicate conference record (*n* = 1). Twenty-seven reports were therefore retained at this stage, of which two were the systematic reviews with meta-analysis described below; these were retained as contextual references rather than as included primary studies, leaving 25 primary studies to be carried forward to re-screening. The additional searches of Scopus, IEEE Xplore, PROSPERO, ClinicalTrials.gov, and WHO ICTRP, conducted on 5 August 2026, retrieved 182 records: Scopus (*n* = 126), IEEE Xplore (*n* = 9), PROSPERO (*n* = 24), ClinicalTrials.gov (*n* = 10), and WHO ICTRP (*n* = 13). The 47 registry records comprised review protocols and trial registrations rather than completed primary studies; none met the eligibility criteria, and no unpublished or ongoing study reporting extractable microstate parameters in an AD-spectrum population was identified. Of the 135 bibliographic records, 29 duplicated records already screened, leaving 106 unique records for title and abstract screening. Ninety-six were excluded at this stage, principally for non-AD-spectrum populations, and 10 full texts were assessed. Six were excluded (three reporting no group-level microstate parameters, two lacking a healthy-control comparator, and one narrative review), and four met all eligibility criteria and were added to the synthesis. The Web of Science Core Collection search, also conducted on 5 August 2026, retrieved 145 records, of which 30 corresponded to records or studies already identified through the preceding searches. Screening the remainder identified four records requiring full-text assessment; all four were excluded, one because the sample was defined by cognitive screening scores alone without formal diagnostic criteria, and three because no group-level microstate parameters were reported. This search therefore identified no additional eligible studies. A final total of 26 primary studies were included in the qualitative synthesis. Two systematic reviews with meta-analyses, Das et al. (2026) [30] and Piton et al. (2026) [31], were identified by the same search but were not counted among the included primary studies; to avoid double counting of overlapping datasets, they were retained as a separate contextual reference layer, appraised using AMSTAR 2 (Supplementary Table S4) and used only to benchmark the direction and magnitude of the primary-study findings. Inter-rater reliability was high at both stages: κ = 0.81 for title and abstract screening and κ = 0.87 for full-text eligibility assessment, indicating substantial and almost-perfect agreement, respectively [42]. Interval estimates for κ were not computed, as the stage-level agreement counts were not retained after screening; all screening disagreements were resolved by discussion, with a third reviewer available for adjudication. The 25 primary studies retained following the initial full-text assessment were re-screened against the a priori PICO criteria (Section 2.2).

Three were reclassified as excluded on re-screening, leaving 22 eligible primary studies: one lacked a healthy-control comparator, and two did not report an extractable microstate parameter. As for the category-level exclusions above, these three records are reported by reason rather than cited individually, and are itemized in the footnote to Supplementary Table S1. The 26 included primary studies [32,33,34,35,44,46,47,48,49,50,51,52,53,54,55,56,57,58,59,60,61,62,63,64,65,66] are characterized in Supplementary Table S1; the specific reason for inclusion of each retained study, mapped to the population, index test, comparator, and outcome criteria, is documented in the right-hand column of Supplementary Table S1. The complete study selection process is depicted in the PRISMA 2020 flow diagram (Figure 2) [37].

Included studies were published between 1997 and 2026, with sample sizes ranging from 28 to 945 participants across individual studies. The modified K-means algorithm [22] was the most widely applied microstate segmentation method, with a smaller proportion of studies employing the atomize and agglomerate hierarchical clustering (AAHC/TAAHC) algorithm [40]. Most studies identified the four canonical microstate classes (A, B, C, D) [21]; one study reported a five-class solution, two applied a seven-class solution, one reported both four- and seven-class solutions, and four did not report the number of classes extracted. Methodological quality of primary studies was evaluated using the Newcastle–Ottawa Scale adapted for cross-sectional studies (NOS-CS) [43]. A comprehensive summary of included study characteristics is presented in Supplementary Table S1.

Sensitivity analysis using the original eligibility criteria. Reapplying the a priori list of diagnostic frameworks (NIA-AA 2011 or 2018, DSM-5, and ICD-10 or ICD-11 only) to the 26 included studies, 13 studies would have been ineligible, either because the framework recorded for them is one of those added by the amendment or because no validated framework was explicitly named: Strik et al. (1997) [51], Dierks et al. (1997) [50], Stevens and Kircher (1998) [57], Nishida et al. (2013) [56], Tait et al. (2020) [44], Lin et al. (2021) [58], Bejia et al. (2023) [60], Nobukawa et al. (2024) [62], Wan et al. (2024) [46], Tarailis et al. (2025) [64], Johansson et al. (2025) [63], Hou et al. (2025) [49], and Lv et al. (2026) [52]. The remaining 13 studies would have been retained. The study-by-study classification, with the framework recorded for each study, is given in Supplementary Table S7.

The principal findings at the MCI and AD dementia stages are preserved under the original criteria. Every primary study cited in Section 3.3 in support of the microstate A, C, and D findings at those stages Musaeus et al. (2019) [53], Musaeus et al. (2020) [33], Smailovic et al. (2019) [35], Lassi et al. (2023) [32], Lian et al. (2021) [54], Yan et al. (2024) [34], and Schumacher et al. (2020) [55], the last applying the NIA-AA 2011 dementia criteria of McKhann et al. [10] is retained, and no direction of effect at either stage is reversed. Two studies cited in the results would be lost: Nishida et al. (2013) [56], one of the four studies contributing null contrasts (Section 3.3.4), and Tait et al. (2020) [44], the source of the sequence-complexity and classification findings discussed in Section 3.3.3 and Section 4.2.3. Of the three shared-cohort clusters defined in Section 2.7, the OpenNeuro cluster would retain two of its four publications and continue to contribute a single vote, whereas the Strik and Dierks companion cohort and the single-study BrainLat cluster would be lost entirely. The number of independent samples contributing to individual cells of Table 1 would therefore fall, and the synthesis would rest on a narrower base, but its direction would not change.

The SCD stage is affected differently and more substantially. The SCD group of Lassi et al. (2023) [32], which supplies the only SCD-versus-control contrast counted in Table 1, was defined using the SCD-I research criteria, one of the frameworks added by the amendment. Under the original list, that contrast would not have been eligible, and the SCD stage would have contributed no counted comparison at all. The amendment was therefore necessary if the review was to address the SCD stage as intended; equally, this result shows how narrow the SCD evidence base is, a point returned to in Section 4.2.1 and Section 5. The second amendment, relaxation of the comparator requirement from age- and sex-matched to age-matched controls, altered no inclusion decision, since no study was included on the basis of that relaxation that would not also have met the original requirement had sex distribution been reported (Section 2.1).

### 3.2. Study Characteristics and Quality Assessment

#### 3.2.1. Population and Diagnostic Criteria

The studies recruited adult participants aged 50 years or older, with mean ages ranging from approximately 60 to 80 years across diagnostic groups. Clinical diagnoses were established predominantly in accordance with NIA-AA 2011 criteria for MCI and AD dementia [9,10], with a subset of studies utilizing DSM-5 or ICD-10/ICD-11 classification systems. More recent studies applied the updated NIA-AA 2018 biological framework [4], which redefines AD based on AT(N) biomarker profiles rather than clinical symptom expression alone [4,5]; these criteria have since been further revised [39], although no included study applied the 2024 revision. Participants were stratified across four clinical stages: neurologically healthy control, SCD, MCI, and AD dementia. Cognitive severity was most frequently quantified using the MMSE. Per-group scores for every included study are tabulated in Supplementary Table S1. Twenty-two of the 26 studies reported a global cognitive measure: 17 reported the MMSE directly, and five used the MoCA; four reported neither. Across the 17 studies reporting the MMSE, group mean scores spanned 27.5–30.0 in healthy control groups, 24.3–27.6 in MCI groups, and 15.3–26.3 in AD dementia groups, the last reflecting the wide range of dementia severity sampled across the evidence base. Because the instruments are not interchangeable, MoCA-based studies are reported separately in Supplementary Table S1 with the instrument identified, rather than being converted to MMSE-equivalent scores.

#### 3.2.2. EEG Acquisition Parameters

Considerable methodological heterogeneity was observed in EEG acquisition parameters across included studies. The median electrode count was 31, with a range spanning from 19 to 256 electrodes. Average artefact-free resting-state recording durations ranged from approximately 2.5 to 22 min. Most studies employed eyes-closed resting-state paradigms with a bandpass filter range of 1–40 Hz; a minority applied narrower filter settings of 2–20 Hz. All included studies used resting-state EEG paradigms, consistent with the eligibility criteria specified in Section 2.2. Notably, although several studies employed high-density arrays of 64 channels or more, and one used up to 256 channels, every included study derived microstates from sensor-level scalp topographies; no included study computed microstates at the source level using a distributed inverse solution. Source-level microstate analysis has been proposed as a means of resolving the cortical generators underlying each topography and of reducing the volume-conduction ambiguity inherent to sensor-space clustering [26], and its absence from the current evidence base represents a methodological gap rather than a negative finding.

#### 3.2.3. Quality Assessment

Methodological quality varied across the included primary studies, with a roughly even split between moderate quality (11/26 studies, 42.3%), accumulating 4–6 stars on the NOS-CS, and high quality (15/26 studies, 57.7%; 7–10 stars) [43]. The most common sources of methodological limitation included the absence of correction for multiple comparisons across the four microstate classes, inadequate reporting of artefact rejection procedures, and absence of blinded outcome assessment. No included primary studies were rated as low quality (0–3 stars). The mean NOS-CS score across the 26 primary studies was 6.69/10. Per-study NOS-CS domain scores and quality grades are presented in Supplementary Table S2. At the domain level, the selection domain was the principal source of variation (Supplementary Table S2); comparability was frequently limited to age matching without adjustment for additional confounders, and the outcome domain was uniformly constrained by the absence of analyst blinding, which was unmet in all studies. On formal appraisal using AMSTAR 2, the two contextual meta-analyses were rated differently. Das et al. (2026) [30] met all seven critical domains and was rated as providing moderate overall confidence, its weaknesses being non-critical (data extraction not stated to be duplicated; funding of the included studies not reported). Piton et al. (2026) [31] was rated as providing critically low overall confidence, reflecting five critical flaws: the absence of a registered protocol, of a list of excluded studies, of any risk-of-bias appraisal of the included studies, of any consideration of risk of bias when interpreting the pooled estimates, and of any assessment of publication bias. Item-level judgements and the basis for each are reported in Supplementary Table S4. This difference is relevant to interpretation: the pooled estimates drawn from Piton et al. [31] are used here only as directional benchmarks and should be read with corresponding caution, which reinforces the decision to treat both reviews as contextual references rather than as included evidence. The two contextual meta-analyses [30,31] were not incorporated as included primary evidence, and their pooled samples were not combined with the primary-study synthesis. Both were instead formally appraised using AMSTAR 2, with domain-level confidence judgements reported in Supplementary Table S4, and their pooled estimates are used only to benchmark the direction and magnitude of the primary-study findings reported below.

### 3.3. EEG Microstate Findings Across the AD Continuum

The primary findings are organized by clinical stage along the AD continuum SCD, MCI, and AD dementia and by microstate class (A, B, C, D). Pooled effect estimates from the two contextual meta-analyses [30,31] are reported separately, as external benchmarks for the primary-study synthesis rather than as pooled evidence generated by this review; they were not combined with primary data. A summary of effect directions related to healthy controls across all included studies is presented in Table 1.

Effect directions are based on the synthesis of the 26 primary studies. References [30,31] denote the two contextual meta-analyses, whose pooled estimates are shown for benchmarking only and were not combined with the primary studies. Cell entries show the direction of change relative to HC (↑ increased, ↓ decreased, ns no significant difference) and, where a pooled random-effects estimate is available from the contextual meta-analyses, the Hedges’ g effect size with its 95% confidence interval; “ns” denotes a non-significant pooled effect. Study-level standardized effect sizes were not uniformly reported across the primary studies. k is the number of independent participant samples contributing to that cell, counted after applying the shared-cohort clustering rule described in Section 2.7; publications analyzing a common cohort contribute a single sample. The direction column gives the number of independent samples reporting an increase, a decrease, or no significant difference relative to healthy controls. No composite consistency grade is assigned: the previous high/moderate/low classification combined direction concordance, effect magnitude, precision, and study quality into a single unvalidated label, and has been replaced by the underlying quantities, reported directly. Pooled estimates from the two contextual meta-analyses [30,31] appear in the final column for external comparison only; they were not generated by this review and were not combined with the primary-study data. Estimates from both meta-analyses are given wherever each provides one, and cells marked discordant are those in which the two reach different conclusions on statistical significance. An asterisk denotes a pooled estimate whose 95% confidence interval excludes the null; ns denotes a non-significant pooled estimate; “Not estimated” indicates that neither meta-analysis pooled that comparison. Smailovic et al. (2019) [17], although it contributes the largest SCD cohort in the evidence base, does not contribute to these counts: that study fitted healthy-control grand-mean maps to the patient recordings and compared microstate parameters between the SCD, MCI and AD groups only, so no parameter contrast against healthy controls is available from it. Its contribution is topographic (classes A, C and D differ from controls) and takes the form of a gradient across patient groups; both are described in Section 3.3 rather than counted here. Null results are counted: where a study measured a parameter for a given class and stage and reported no significant difference from controls, it contributes an ns vote rather than being omitted. Because primary reports emphasize positive findings, the ns counts should be regarded as a lower bound; a study is entered as ns only where the source text or tables make the null comparison explicit. Every direction assignment was checked against the primary report in which it originated, and the cell-by-cell attribution, with the location in that report supporting each assignment, is given in Supplementary Table S9. Seven reports were re-examined in full for this purpose; eight assignments were corrected, and two studies were withdrawn from the class-level counts because their microstate classes cannot be mapped onto the four canonical classes: Teipel et al. (2021) [59], which labels its microstates 1–4 and assigns no canonical labels, and Johansson et al. (2025) [63], which uses a seven-class solution in mutation-defined groups. Assignments drawn from reports not re-examined are identified as such in Supplementary Table S9. ‡ The source meta-analysis reports an identical 95% confidence interval, test statistic, and *p*-value for the duration and occurrence analyses of microstate D in MCI; the point estimate is therefore reported without an interval (see Section 3.3.2).

#### 3.3.1. Findings in Subjective Cognitive Decline

Evidence for EEG microstate alterations at the SCD stage is limited but emerging. The most methodologically rigorous primary study to examine the SCD stage was Lassi et al. (2023), which recruited participants across SCD, MCI, and HC groups drawn from the same multicenter cohort [32]. That study identified the most prominent microstate alterations at the SCD stage in microstate C, reporting reduced duration and coverage of this class relative to HC, a pattern interpreted as reflecting early disruption of the DMN [26,32,54]. Critically, these alterations in microstate C temporal dynamics became more pronounced at the MCI stage, suggesting a graded and progressive degradation in DMN organization along the continuum [32].

A further finding of substantial clinical significance was the association between microstate C topography and CSF biomarker profile observed by Lassi et al. (2023) [32]. Participants with an AD-like CSF biomarker signature characterized by reduced Aβ42 and elevated phosphorylated tau exhibited significant differences in the spatial topography of microstate C relative to those with a normal CSF profile, even at the SCD stage [32]. This finding provides preliminary empirical support for a neurophysiological link between microstate C dynamics and underlying amyloid pathology at the preclinical stage of the AD continuum. It should be noted that no dedicated SCD-only cohort study employing EEG microstate analysis with full AT(N) biomarker integration was identified in the present review; the SCD-specific microstate contrasts summarized above are derived principally from the multigroup study of Lassi et al. (2023) [32]; a second cohort, Smailovic et al. (2019) [35], also included a large SCD subgroup (*n* = 210) spanning the continuum, representing a significant gap in the current evidence base that is discussed further in Section 4 [5,32].

#### 3.3.2. Findings in Mild Cognitive Impairment

The MCI was the most extensively studied clinical stage across the identified literature. The two contextual meta-analyses, Das et al. (2026) [30] and Piton et al. (2026) [31], which are not counted among the included primary studies (Section 2.7), both reported statistically significant pooled microstate alterations in MCI relative to HC. Their effect estimates are reported here only as external benchmarks against which the primary-study synthesis is compared, and not as independent evidence, because both pool a substantially overlapping set of the same primary studies.

With respect to microstate A, pooled estimates from Piton et al. (2026) [31], which incorporated six eligible MCI studies comprising 293 MCI patients and 324 HC (total N = 617), identified significant increases across all three principal temporal parameters: duration (Hedges’ g = 0.398, 95% CI [0.163, 0.632], *p* = 0.007), occurrence (Hedges’ g = 0.369, 95% CI [0.152, 0.586], *p* = 0.007), and coverage (Hedges’ g = 0.462, 95% CI [0.262, 0.662], *p* = 0.002). These estimates indicate that MCI patients exhibited significantly longer, more frequent, and proportionally greater coverage of microstate A relative to HC. Estimates of similar direction and magnitude were reported by Das et al. (2026) [30], which found a significant increase in microstate A occurrence in MCI (Hedges’ g = 0.40, 95% CI [0.07, 0.74]). Because the two meta-analyses draw on a substantially overlapping set of primary studies, this agreement should not be interpreted as independent corroboration; the estimates were, however, also consistent with individual primary-study findings from Musaeus et al. (2019) [53] and Lassi et al. (2023) [32].

Conversely, microstate D was reduced in MCI in the pooled estimates of both contextual meta-analyses, which draw on a substantially overlapping set of primary studies and are therefore not independent of one another (Supplementary Table S6). At the primary-study level, the evidence is considerably thinner: only two independent samples contribute a microstate D contrast at the MCI stage, one reporting a reduction and one reporting no significant difference (Table 1). Pooled estimates from Piton et al. (2026) [31] indicated significant decreases in microstate D duration (Hedges’ g = −0.306, 95% CI [−0.500, −0.111], *p* = 0.010), occurrence (Hedges’ g = −0.190; see note below), and coverage (Hedges’ g = −0.319, 95% CI [−0.454, −0.184], *p* = 0.002). We note an inconsistency in the source report: Piton et al. give an identical confidence interval, test statistic, and *p*-value (95% CI [−0.500, −0.111], z|t = −4.04, *p* = 0.010) for both the duration and the occurrence analyses of microstate D, although the point estimates differ (g = −0.306 and g = −0.190, respectively). These cannot both be correct. We therefore report the point estimates as published and do not reproduce the duplicated interval for the occurrence analysis; the direction of effect is supported by the corresponding forest plot in the source. Das et al. (2026) [30] reported a significant reduction in microstate D duration in MCI (Hedges’ g = −0.26, 95% CI [−0.48, −0.04]), of comparable direction and magnitude to the Piton et al. estimate; as noted above, the overlap between the two meta-analyses means this agreement is not independent corroboration (Supplementary Table S6). As microstate D is functionally associated with the frontoparietal attention network [31], its reduction in MCI may reflect early impairment of attentional allocation and executive control processes.

Neither Das et al. (2026) [30] nor Piton et al. (2026) [31] identified statistically significant pooled effects for microstate B or microstate C at the MCI stage. However, at the level of individual primary studies, Lassi et al. (2023) [32] reported reduced microstate C duration and coverage in their MCI subgroup, and Musaeus et al. (2019) [53] observed significantly increased occurrence (*p* = 0.028) and coverage (*p* = 0.010) of microstate A but no significant difference in microstate C at either the MCI or the AD stage. That study recruited 38 healthy controls, 27 patients with MCI and 17 with AD, of whom 37, 25 and 15, respectively, contributed analyzable recordings, at two Danish memory clinics. On re-examination of the primary reports, the microstate C reduction at the MCI stage rests on Lassi et al. (2023) [32] alone: of the six independent samples contributing a microstate C contrast at this stage, one reports a decrease, two an increase, and three no significant difference (Table 1). Microstate C alterations at the MCI stage are therefore neither consistent in direction nor supported by pooled estimates.

Beyond temporal parameters, Lassi et al. (2023) [32] demonstrated at the primary-study level that MCI was associated with altered microstate transition probabilities and reduced complexity of microstate sequences, reflecting disruption not only in the duration of individual cortical states but also in the sequential organization of state transitions. This finding highlights the additional diagnostic value of microstate syntax analyses beyond conventional temporal parameter reporting.

#### 3.3.3. Findings in Alzheimer’s Disease Dementia

The AD dementia stage was the most extensively studied across the identified literature. Pooled estimates from Piton et al. (2026) [31] were derived from ten eligible AD studies comprising 346 AD patients and 355 HC (total N = 701), while Das et al. (2026) [30] identified 30 eligible studies in total, of which 16 were included in their quantitative synthesis. Because these two meta-analyses were conducted separately but drew on a substantially overlapping pool of primary studies, the agreement between their pooled estimates reflects shared source data as much as reproducibility and does not constitute independent replication; the extent of this overlap is documented in Supplementary Table S6. The consistency observed across the individual primary studies therefore carries the greater evidential weight for EEG microstate alterations in established AD.

Prolongation of microstate A and B duration was the most frequently reported finding at the AD stage, although the direction was not uniform across the contributing samples. Of the seven independent samples contributing a microstate A contrast at this stage, two report an increase and five no significant difference; of the eight contributing a microstate B contrast, three report an increase, none a decrease, and five no significant difference (Table 1). Pooled estimates from Piton et al. (2026) [31] indicated significant increases in microstate A duration (Hedges’ g = 0.440, 95% CI [0.216, 0.663], *p* = 0.002), microstate A coverage (Hedges’ g = 0.468, 95% CI [0.198, 0.738], *p* = 0.004), and microstate B duration (Hedges’ g = 0.399, 95% CI [0.262, 0.537], *p* = 0.0001). Comparable pooled estimates were reported by Das et al. (2026) [30] for microstate A duration (Hedges’ g = 0.41, 95% CI [0.10, 0.72]) and microstate B duration (Hedges’ g = 0.48, 95% CI [0.23, 0.73]). These pooled estimates are closely comparable, but because the two meta-analyses share a substantial proportion of their contributing primary studies (Supplementary Table S6), their agreement cannot be treated as independent verification of microstate A and B prolongation. The prolongation of microstate A, functionally associated with auditory and language processing networks, and microstate B, linked to visual network dynamics, may reflect a compensatory engagement of sensory processing networks as higher-order heteromodal association networks progressively deteriorate.

With respect to microstates C and D, pooled estimates from Piton et al. (2026) [31] indicated significant reductions in microstate C occurrence (Hedges’ g = −0.213, 95% CI [−0.389, −0.036], *p* = 0.022) and microstate D occurrence (Hedges’ g = −0.304, 95% CI [−0.457, −0.151], *p* = 0.002) in AD. Das et al. (2026) [30] reported non-significant pooled effects for microstate C and D duration in AD, attributing this to heterogeneity across primary studies in the measurement and reporting of these parameters. This pooled reduction in microstate C occurrence is not reproduced at the primary-study level. Of the six independent samples contributing a microstate C contrast at the AD dementia stage, two report an increase (Yan et al. 2024 [34]; Lv et al. 2026 [52]), four report no significant difference, and none report a significant reduction (Table 1; Supplementary Table S9). The pooled estimate is therefore the sole source of this direction; a reduction would be consistent with the extensive functional MRI literature documenting DMN disruption as a cardinal feature of AD [26,54,56], but the present synthesis does not support it independently.

At the level of individual primary studies, Yan et al. (2024) [34] examined 56 AD patients and 38 HC with concurrent CSF biomarker assessment and reported that microstate B mean duration and occurrence were significantly correlated with CSF Aβ1-42 levels (*p* < 0.05), and microstate C duration was significantly correlated with CSF Aβ1-40 levels (*p* < 0.05). Microstate parameter changes were additionally associated with Stroop Color Word Test performance and Activities of Daily Living scale scores, indicating that microstate alterations reflect both underlying amyloid pathology and clinically meaningful functional impairment [33]. In the domain of automated classification, Tait et al. (2020) [44] reported at the primary-study level that microstate-derived features outperformed conventional spectral EEG feature sets in a machine-learning classifier, although this finding derives from internal cross-validation in a single small cohort and requires external validation.

#### 3.3.4. Heterogeneity and Inconsistent Findings

Substantial statistical and methodological heterogeneity was observed across included studies. Das et al. (2026) [30] reported I^2^ values exceeding 50% for several microstate parameters in both MCI and AD, attributing this heterogeneity to variability in electrode count, reference scheme, clustering algorithm, minimum microstate duration threshold, and diagnostic classification instruments, factors that precluded a de novo pooled analysis within the scope of the present review. Several individual primary studies reported no statistically significant microstate differences between patient groups and HC. On re-examination of the primary reports, Nishida et al. (2013) [56] reported no significant AD-versus-control difference for any microstate class or parameter, and Musaeus et al. (2019) [53] reported none for microstates B, C, and D at either stage. Schumacher et al. (2020) [55] describe its AD-versus-control comparison as a null finding. Lian et al. (2021) [54] is not a null study, contrary to the characterization in Piton et al. (2026) [31]: it reports microstate B duration (*p* = 0.008) and coverage (*p* = 0.007) significantly increased in AD relative to controls, with its remaining contrasts non-significant. These null findings are not necessarily inconsistent with the broader pattern of evidence, given differences in sample size (some studies included as few as eight participants per group), EEG recording system configurations, and analytical pipeline choices [30,31].

### 3.4. AT(N) Biomarker Correlates of EEG Microstates

Integration of EEG microstate parameters with AT(N) biomarkers was examined in a small subset of identified primary studies. As described in Section 3.3.1, Lassi et al. (2023) [32] reported topographic differences in microstate C associated with an AD-like CSF Aβ42/p-tau profile at both the SCD and MCI stages; this is the earliest stage at which an AT(N)–microstate linkage has been reported in the identified literature, although it rests on a single study and no included study examined a stage earlier than SCD, so the finding marks the limit of where the evidence base has looked rather than an established point of onset. Yan et al. (2024) [34] subsequently demonstrated significant correlations between CSF Aβ1-42 levels and microstate B temporal parameters at the AD dementia stage. No identified study, however, directly mapped microstate parameters onto the complete AT(N) tripartite classification framework proposed by Jack et al. (2018) [4] within a longitudinal, continuum-spanning design. This represents a major gap in the existing evidence base, which is discussed further in Section 4.2.

### 3.5. Summary of Evidence Across the Continuum

The cumulative evidence from the 26 primary studies, benchmarked against the two contextual meta-analyses, is consistent with a graded pattern of EEG microstate differences across clinically defined diagnostic groups. This pattern is inferred from between-group comparisons of separate cohorts rather than from within-individual progression, and the evidence is unevenly distributed across stages, being substantially weaker at the SCD stage than at the MCI and AD dementia stages. At the SCD stage, preliminary evidence from Lassi et al. (2023) [32] indicates early degradation of microstate C temporal dynamics in association with CSF amyloid pathology; the Smailovic et al. (2019) cohort [35] contributed a further 210 SCD participants, although the SCD-specific microstate contrasts derive principally from the former study and no pooled estimate is available. At the MCI stage, the primary-study synthesis, benchmarked against the two contextual meta-analyses, which draw on a substantially overlapping pool of primary studies (Supplementary Table S6), indicates increases across the temporal parameters of microstate A and decreases across those of microstate D, with moderate-to-small effect sizes (Hedges’ g = 0.37–0.46 for microstate A; Hedges’ g = 0.19–0.32 for microstate D). The primary-study evidence underlying these two statements is uneven and not uniform in direction: five independent samples contribute a microstate A contrast at this stage, of which three report an increase, one a decrease, and one no difference, whereas only two contribute a microstate D contrast, one reporting a decrease and one no difference (Table 1). The pooled direction should therefore not be read as replication across samples. At the AD dementia stage, prolongation of microstate A and B duration is the direction most often reported across both contextual meta-analyses and individual primary studies [30,31], accompanied by a reduction in microstate D occurrence, which two independent samples support and none contradict; the corresponding pooled reduction in microstate C occurrence is not reproduced in any individual sample (Table 1). The AD-stage findings are further accompanied by demonstrated correlations between microstate parameters and CSF amyloid levels and functional performance measures in primary studies [34].

Taken together, these findings indicate that EEG microstate parameters, particularly those of microstates A, C, and D, differ at the group level between clinically defined diagnostic groups in a graded fashion and may reflect progressive disruption of large-scale cortical networks. These are cross-sectional group-level differences; they do not establish diagnostic accuracy, early-detection capability, or staging utility for any individual patient, and the parameters are therefore best regarded at present as preliminary neurophysiological correlates rather than as validated biomarkers. Confirmation of this potential awaits standardized, longitudinal, and AT(N)-integrated multimodal studies [4,25,30,31].

## 4. Discussion

### 4.1. Overview of Principal Findings

This systematic review synthesized the available evidence on EEG microstate dynamics in clinically defined SCD, MCI, and AD dementia samples. The principal finding to emerge from the identified literature is that EEG microstate alterations are not uniformly distributed across the continuum but differ in magnitude across diagnostic groups in a graded fashion that is broadly compatible with the hierarchical accumulation of neuropathological burden proposed by the NIA-AA AT(N) framework [4]. It should be emphasized that this gradient is inferred from between-group comparisons of separate cohorts rather than from within-individual progression, and that most contributing samples were defined clinically rather than by biomarker confirmation of AD pathology. Compatibility with the AT(N) framework should therefore be read as descriptive rather than as demonstrated alignment: no included study mapped microstate parameters onto the complete AT(N) tripartite classification (Section 3.4), only four of the 26 studies reported concurrent AT(N) biomarkers at all, and the evidence at the SCD stage rests principally on a single multigroup study. The staging inference is correspondingly weakest at precisely the earliest stage where it would be of greatest clinical value.

The directions most frequently reported, drawn from the narrative synthesis of the 26 primary studies and benchmarked against the pooled estimates of two contextual meta-analyses [30,31] that are not independent of one another (Supplementary Table S6), are prolonged duration and increased coverage of microstate A beginning at the MCI stage, progressive reduction in microstate D across all temporal parameters from MCI through AD dementia, and prolongation of microstate B duration emerging predominantly at the AD dementia stage. Microstate C is the least secure of these. Its reduction at the SCD stage rests on a single contrast, its direction at the MCI stage is discordant across samples, and at the AD dementia stage a pooled reduction in occurrence coexists with two samples reporting an increase and none reporting a decrease (Table 1). A reduction would be consistent with the extensive functional neuroimaging literature documenting DMN disorganization as a cardinal feature of AD [26,54,56]. None of these is a uniform finding. No parameter-by-stage outcome in Table 1 has all of its contributing samples agreeing in direction; the number of independent samples per outcome ranges from none to six; and for microstate D at the MCI stage only two samples contribute. These are therefore the directions most often reported within a small and partly discordant evidence base, and the term replication is not warranted for any of them. These findings are discussed in the context of their neurobiological interpretation, their methodological constraints, and their translational implications.

### 4.2. Stage-Specific Microstate Alterations and Their Neurobiological Interpretation

#### 4.2.1. Subjective Cognitive Decline

EEG microstate evidence at the SCD stage remains sparse. The SCD-specific contrasts summarized here derive principally from Lassi et al. (2023) [32]; the large Smailovic et al. (2019) cohort [35] also included 210 SCD participants spanning the AD continuum, so SCD evidence is not derived from a single study alone. The observation by Lassi et al. (2023) of reduced microstate C duration and coverage in SCD relative to HC, in association with an AD-like CSF biomarker profile, is of considerable theoretical importance, as it locates a microstate disruption within the context of preclinical amyloid pathology [32]. Because this observation derives from a single multigroup study, it should not be read as establishing that microstate C degradation is the earliest detectable neurophysiological change on the continuum; no study in the identified literature compared candidate markers head-to-head for time of first detectability. Microstate C has been consistently linked to the functional dynamics of the DMN [26,27,54], and its degradation at the SCD stage is conceptually consistent with the earliest measurable DMN disruptions reported in amyloid-PET-positive cognitively unimpaired individuals [54,56]. However, the absence of any dedicated SCD cohort study with concurrent AT(N) biomarker profiling and longitudinal follow-up renders these observations preliminary. This represents perhaps the most critical evidence gap identified by this review, given that effective disease-modifying intervention is most likely to succeed at the preclinical stage [4,9].

#### 4.2.2. Mild Cognitive Impairment

The MCI literature is more extensive and internally consistent than that at the SCD stage. The Musaeus et al. (2019) Nordic multicenter primary study [53] was among the first to show that elevated occurrence and coverage of microstate A were detectable in MCI relative to HC. A subsequent extended multicenter study [33] confirmed these alterations in a larger cohort of 135 HC, 117 MCI, and 117 AD participants recruited from six memory clinics. That study also reported that microstate features classified HC from MCI with an accuracy of 58.7%, and HC from AD with an accuracy of 69.8%. These primary-study findings were subsequently evaluated alongside pooled estimates from Piton et al. (2026) [31], who synthesized 12 eligible studies and reported significant increases across all three temporal parameters of microstate A duration (Hedges’ *g* = 0.398), occurrence (*g* = 0.369), and coverage (*g* = 0.462) alongside significant decreases in all temporal parameters of microstate D.

Das et al. (2026) [30] reported estimates of comparable direction and magnitude, although the overlap in their contributing primary studies means this agreement is not independent corroboration (Supplementary Table S6). The reductions in microstate D functionally associated with the frontoparietal attention network may reflect early impairment of attentional resource allocation and executive function, which are among the earliest cognitive domains affected in amnestic MCI [27]. Taken together, these convergent findings across primary studies and contextual meta-analyses position MCI as the stage at which microstate alterations are most often detected. This falls well short of statistical robustness or of reproducibility across independent datasets: effect sizes are moderate-to-small, few independent samples contribute to each outcome, and their directions are not uniform (Table 1). Large-sample validation in independent, biomarker-stratified cohorts would be required before these parameters could be treated as candidate neurophysiological biomarkers.

#### 4.2.3. Established Alzheimer’s Disease Dementia

The most extensive and convergent body of evidence pertains to established AD dementia. Das et al. (2026) [30], encompassing 30 eligible studies of which 16 were included in quantitative synthesis, reported prolonged microstate A and B durations and reduced microstate D duration as the most frequently recurring findings among the studies it pooled, albeit with substantial inter-study heterogeneity (I^2^ exceeding 60% for most parameters), a level of heterogeneity that precluded a de novo pooled analysis within the scope of the present review. Piton et al. (2026) [31], which included ten eligible AD primary studies comprising 346 AD patients and 355 HC, reported estimates of comparable direction and magnitude; given the overlap between the two meta-analyses in their contributing primary studies (Supplementary Table S6), this agreement is reported as consistency rather than as independent verification of microstate A and B prolongation. From a network perspective, microstate A has been functionally linked to auditory and language processing networks, while microstate B is associated with visual network dynamics [27]; their simultaneous prolongation in AD may reflect a compensatory engagement of unimodal sensory processing networks as higher-order heteromodal association networks deteriorate, a pattern analogous to the posterior-to-anterior cortical atrophy gradient observed on structural MRI.

Yan et al. (2024) [34] reported a correlation between CSF Aβ1-42 levels and microstate B temporal parameters. This is the most direct empirical support currently available for a link between amyloid burden and microstate prolongation in AD, and it merits replication in well-powered, biomarker-stratified cohorts. Tait et al. (2020) [44] reported that reduced Lempel-Ziv complexity of microstate transition sequences classified AD from HC with sensitivity and specificity exceeding 80% when combined with spectral slowing measures, and that this metric showed preliminary predictive value for MCI-to-AD conversion over a four-year follow-up. This finding is notable because it extends the informational content of microstate analysis beyond temporal parameters to the sequential organization of cortical state transitions, and it warrants prospective validation.

### 4.3. Relationship to AT(N) Biomarkers and Cognitive Measures

A key gap in the current literature and a central motivation for this review is the limited integration of EEG microstate parameters with the NIA-AA AT(N) biomarker framework [4]. Across all included primary studies, only four reported microstate parameters alongside concurrent AT(N) biomarkers (Smailovic et al. 2019 [35], Lassi et al. 2023 [32], Lin et al. (2021) [58], and Yan et al. (2024) [34]), and only Yan et al. (2024) [34] incorporated a comprehensive multi-marker CSF panel encompassing Aβ1-42, Aβ1-40, phosphorylated tau, total tau, and neurofilament light chain alongside clinical and functional assessments. This renders conclusions about whether individual microstate parameters independently track amyloid burden (A), tauopathy (T), or neurodegeneration (N), or whether they reflect a composite of pathophysiological processes, necessarily speculative at this stage. Correlations between microstate parameters and cognitive performance measures including MMSE, MoCA, and composite neuropsychological scores were reported in a minority of included primary studies, with inconsistent directionality across studies and parameters, underscoring the need for adequately powered studies designed to detect microstate cognition relationships as primary rather than secondary exploratory endpoints. The incorporation of emerging blood-based biomarkers, particularly plasma phosphorylated tau-217 and amyloid ratio indices [8], alongside EEG microstate assessment in longitudinal SCD and MCI cohorts represents a methodological priority for the next generation of studies in this field, as it would permit the mapping of microstate trajectories onto the continuous AT(N) staging framework in a manner not yet achievable from the existing cross-sectional evidence base [4,6]. This limited biomarker coverage carries an important interpretive consequence: in the remaining 18 studies, SCD and MCI groups were defined by clinical and cognitive criteria alone, without confirmation of underlying amyloid or tau pathology. Such groups are etiologically heterogeneous, and only a proportion of clinically defined SCD and MCI cases ultimately prove to have AD pathology. The stage labels used throughout this review therefore denote clinical syndromes rather than biologically confirmed positions on the AD continuum, and the observed microstate gradient cannot be attributed specifically to AD pathophysiology on the present evidence.

### 4.4. Methodological Heterogeneity and Its Implications

A significant and recurring finding of this review is the extent of methodological variability across included studies, which collectively precluded a de novo quantitative meta-analysis and necessitated a structured narrative synthesis as the primary approach. Differences in EEG channel density (ranging from 19 to 256 electrodes), preprocessing pipelines, segmentation algorithms (modified K-means [22], AAHC [40], Topographic AAHC), reference schemes, number of microstate classes (4, 5, or 7), and frequency bandpass filter settings (broadband versus sub-band) collectively generate a mosaic of findings that resists straightforward quantitative synthesis [21,24]. Michel and Koenig (2018) [21] identified these sources of variance as fundamental obstacles to cross-study comparability in EEG microstate research, and Michel and Bréchet (2026) [26] have more recently called for field-wide standardization of microstate preprocessing and reporting conventions as a prerequisite for clinical translation.

The MICROSTATELAB toolbox for EEGLAB [25] provides an open-source analytical platform that could facilitate methodological harmonization across clinical research settings, though its adoption within the dementia EEG literature has thus far been uneven. The absence of a dedicated EEG microstate reporting standard analogous to CONSORT or STROBE for this specific application domain represents an important unmet need that the field has yet to address. Critically, normative reference data for microstate parameters across the healthy ageing spectrum are now available, synthesized by Zanesco (2024) [29] across 93 studies and 6583 participants, a resource that was unavailable to most studies reviewed here, and that provides an essential benchmark against which clinically significant deviations can now be assessed prospectively.

### 4.5. Limitations of Included Studies and of This Review

Several important limitations must be acknowledged in interpreting the evidence synthesized in this review. First, many, though not all, included primary studies employed cross-sectional designs (longitudinal follow-up was reported by Tait et al. (2020) [44], which fundamentally limit causal inference and preclude determination of whether microstate alterations preceded, coincide with, or follow conventional cognitive and biomarker milestones along the AD continuum. In addition, not all included publications represent independent samples: Wan et al. (2024) [46], Yang et al. (2024) [47], Chang (2025) [48], and Hou et al. (2025) [49] analyze the same publicly available OpenNeuro EEG dataset, and Dierks et al. (1997) [50] and Strik et al. (1997) [51] are companion reports of a single cohort. Convergent findings among these publications therefore reflect analytical diversity applied to shared data rather than independent replication, and they were treated as non-independent in the synthesis, each cluster contributing a single vote to the direction-of-effect tally and a single unit to k, as specified in Section 2.7; two further studies that had drawn on the same OpenNeuro dataset were removed during re-screening (Section 3.1). Lv et al. (2026) [52] likewise draws on the shared BrainLat cohort. Second, sample sizes across individual primary studies were generally modest, with a median of fewer than 50 participants per group, which is insufficient to support subgroup analyses by biological sex, APOE ε4 carrier status, or comorbidity burden, all of which may substantially modulate EEG microstate parameters in the ageing population [17,18]. Third, diagnostic criteria varied across studies, with some applying NIA-AA 2011 criteria [9,10] and others utilizing DSM-5 or ICD-10 frameworks, introducing potential heterogeneity in case ascertainment that may partially explain inconsistent findings at the parameter level. Fourth, this review is itself limited by the restriction to English-language full-text publications and to studies reporting quantitative microstate parameters, exclusions that may have omitted methodologically relevant evidence, particularly from research centers in East Asia that have contributed substantially to the EEG biomarker literature in recent years [49]. Additionally, although the search was expanded in response to peer review to five bibliographic databases (PubMed/MEDLINE, EMBASE, Scopus, IEEE Xplore and Web of Science Core Collection) together with three registries, PsycINFO was not searched because it is not held under the authors’ institutional subscription; the institution subscribes to PsycARTICLES, a full-text collection of American Psychological Association journals, which is not an equivalent abstracting and indexing resource. Its omission is unlikely to have materially affected retrieval, as the Web of Science search returned no eligible study that had not already been identified and the registries yielded no eligible unpublished or ongoing study, but it remains a limitation that future updated searches should address. Fifth, only two included primary studies reported inter-rater reliability metrics for EEG preprocessing decisions, and none explicitly reported blinding of microstate analysts to clinical diagnosis, a potential source of ascertainment bias that future studies should address as a minimum reporting standard. Sixth, the present review did not conduct a de novo meta-analysis; accordingly, the quantitative effect size estimates reported in Section 3 are derived from two contextual meta-analyses [30,31] rather than from an independent pooling of primary data, which represents a methodological limitation that future reviews with broader primary-study pools should seek to address. Seventh, the individual-study data available for synthesis were limited: although group sizes, diagnostic criteria, analytical pipelines, and the direction of each key finding are tabulated for every study in Supplementary Table S1, the majority of primary studies did not report group-level means with dispersion statistics or a standardized effect estimate with its confidence interval, so study-level effect estimates could not be presented for all outcomes (documented per outcome in Supplementary Table S5). Availability of these statistics was judged during extraction at the level of the evidence base as a whole rather than recorded study by study, which is itself a limitation of the extraction process; the analytical parameters that bear on cross-study commensurability, segmentation algorithm, number of microstate classes extracted, and the form in which parameters are reported are tabulated for every study in Supplementary Table S8. The quantitative effect sizes shown in Table 1 are therefore pooled benchmarks drawn from the two contextual meta-analyses rather than estimates computed for each study. Eighth, risk of bias arising from missing results was not assessed quantitatively. Funnel-plot asymmetry and regression-based tests such as Egger’s test require a pooled analysis with a sufficient number of comparable estimates and were therefore not applicable to the present narrative synthesis; the possibility that studies reporting null microstate findings are underrepresented in the published literature cannot be excluded, and the search-stage measures adopted to mitigate this (reference-list hand-searching and the absence of any language restriction, Section 2.3) reduce but do not eliminate this risk. Ninth, confidence in the body of evidence was appraised narratively rather than through a formal framework: no GRADE assessment was undertaken, and the descriptors of consistency reported in Table 1 reflect direction concordance, effect magnitude and precision, the number of contributing studies, and NOS-CS quality, but should not be read as formal certainty ratings. Finally, the quality of available AT(N) biomarker data across included studies was insufficient to permit any formal meta-regression of microstate parameters against continuous amyloid or tau indices, leaving the pathological specificity of individual microstate parameters unresolved [4].

### 4.6. Clinical Translation and Future Directions

Any clinical translation of EEG microstate parameters remains a distant prospect, and the present evidence base does not support their use as diagnostic or staging tools. Before EEG microstate parameters can be considered for integration into clinical diagnostic or monitoring pathways, several conditions must be satisfied: prospective longitudinal validation in large cohorts enriched for preclinical and prodromal AD using AT(N) biomarker stratification [5,44]; establishment of sensitivity and specificity thresholds relative to established biomarkers such as amyloid-PET and CSF phosphorylated tau [6,8]; demonstration of test-retest reliability under real-world clinical recording conditions, including systems with as few as 19 electrodes in routine outpatient EEG suites [17,21]; and the development of automated, pipeline-agnostic microstate analysis tools that have been independently validated across multiple sites and EEG systems [25].

The inherent advantages of EEG microstates as a biomarker modality, principally their non-invasiveness, low cost, accessibility in resource-limited healthcare settings, and millisecond-scale temporal resolution, make this approach worth investigating further for future cognitive screening and monitoring applications [17,18], although no included study evaluated microstate parameters in a screening context or against a prespecified diagnostic threshold. The classification accuracies reported by a minority of studies derive from internal cross-validation within single cohorts, without external validation or reporting in accordance with diagnostic-accuracy standards, and therefore do not constitute evidence of diagnostic performance. Integration with machine-learning frameworks, exemplified by Tait et al. (2020) [44], in which microstate features are combined with spectral and connectivity measures, may further enhance predictive accuracy. However, the risk of overfitting inherent in small clinical sample sizes warrants cautious interpretation of reported classification metrics until robust external validation across independent, demographically diverse, and biomarker-stratified cohorts has been achieved [30,31]. Multimodal integration of EEG microstate parameters with emerging fluid biomarkers, neuroimaging, and digital cognitive assessments represents the most promising near-term avenue for establishing the clinical utility of this approach along the full AD continuum [4,25,30].

## 5. Conclusions

This systematic review synthesized evidence on resting-state EEG microstate dynamics in clinically defined SCD, MCI, and AD dementia samples. Its principal distinguishing contribution is the explicit inclusion of the SCD stage, which previous syntheses in this field have not covered: existing reviews and both contextual meta-analyses [30,31] address only the MCI and AD dementia contrasts. The cumulative findings, drawn from the 26 primary studies and benchmarked against two published meta-analyses used as contextual references [30,31], describe a graded pattern of group-level differences in microstate parameters that is compatible with progressive deterioration of large-scale cortical network organization [5,6], but that is derived almost entirely from cross-sectional comparisons between separate cohorts. At the SCD stage, reduced microstate C temporal parameters were reported in association with CSF amyloid-β pathology [32,35], implicating default mode network disruption before objective cognitive impairment is demonstrable. This is the finding of greatest potential value in the present synthesis and the least secure: it derives principally from one multigroup study, with additional SCD participants contributed by a single further cohort, and no pooled estimate is available. The SCD stage is the earliest point on the continuum at which any contrast was available, but this reflects where the available studies looked rather than where neurophysiological change begins: a single study supplies the only SCD-versus-control comparison, no included study examined an earlier stage, and no study compared candidate markers for time of first detectability. Establishing whether microstate C degradation is a reproducible preclinical signature is therefore the clearest priority arising from this review. Beyond the SCD stage, prolonged microstate A duration and coverage, together with reduced microstate D temporal parameters, represent the most frequently reported alterations at the MCI stage, on few independent samples whose directions are not uniform [30,31]. At the AD dementia stage, prolongation of microstate B duration and reduction in microstate D occurrence are the directions most often reported, each supported by more than one independent sample; the pooled reduction in microstate C occurrence at this stage is reproduced by no individual sample and is not treated here as an established feature of the signature of established disease [30,31,34]. Preliminary evidence from the SCD stage suggests that disruption of microstate C dynamics functionally linked to the default mode network [26,54] is detectable at the SCD stage and co-occurs with preclinical amyloid pathology as characterized by CSF biomarker profiling [32]. This rests on one multigroup study and a second cohort contributing SCD participants, and no pooled estimate is available; it therefore identifies a priority for replication rather than an established earliest marker.

These findings carry several important implications. EEG microstate parameters represent temporally sensitive, non-invasive, and low-cost indices of large-scale network disorganization that confer practical advantages over established AT(N) biomarker modalities in resource-limited settings [17,18,21,27]. The presence of measurable microstate differences in MCI samples makes this approach worth evaluating further alongside established biomarker-based strategies [9,10], although no included study assessed microstate parameters as a screening or diagnostic test against a reference standard, so no claim of diagnostic or early-detection utility can be supported at present. The correlations identified between microstate parameters and CSF amyloid indices provide an initial empirical basis for situating microstate dynamics within the AT(N) framework, although substantial longitudinal and multimodal validation remains necessary before clinical application can be considered [4,34].

The most immediate research priority is the integration of EEG microstate assessment with emerging blood-based biomarkers, particularly plasma phosphorylated tau-217 and amyloid ratio indices [6,8] within prospective longitudinal cohorts enriched for preclinical and prodromal AD, alongside field-wide adoption of standardized analytical pipelines [25] and normative reference frameworks [29]. Realizing the clinical potential of EEG microstate dynamics requires a coordinated program of standardized, longitudinal, and biomarker-integrated research that the field is now methodologically equipped to pursue [25,29,30,31].

## Figures and Tables

**Table 1:** Summary of EEG microstate parameter changes across clinical stages relative to HC.

Microstate	Stage vs. HC	k (Independent Samples)	Direction Across Samples (↑/↓/ns)	Parameters Reported	Pooled Estimates from Contextual Meta-Analyses
A	SCD	0	0/0/0	Duration, occurrence, coverage	Not estimated
A	MCI	5	3/1/1	Duration, occurrence, coverage	Piton: dur 0.40 [0.16, 0.63] *, occ 0.37 [0.15, 0.59] *, cov 0.46 [0.26, 0.66] * · Das: dur 0.21 [−0.03, 0.45] ns, occ 0.40 [0.07, 0.74] * discordant for duration
A	AD	7	2/0/5	Duration, occurrence, coverage	Piton: dur 0.44 [0.22, 0.66] *, cov 0.47 [0.20, 0.74] * · Das: dur 0.41 [0.10, 0.72] *, occ 0.13 [−0.11, 0.37] ns
B	SCD	0	0/0/0	Duration, occurrence, coverage	Not estimated
B	MCI	6	2/1/3	Duration, occurrence, coverage	Piton: dur 0.25 [−0.25, 0.75] ns · Das: dur 0.20 [−0.08, 0.47] ns, occ 0.04 [−0.24, 0.31] ns
B	AD	8	3/0/5	Duration, occurrence, coverage	Piton: dur 0.40 [0.26, 0.54] * · Das: dur 0.48 [0.23, 0.73] *, occ 0.21 [−0.10, 0.53] ns
C	SCD	1	0/1/0	Duration, coverage, topography	Not estimated
C	MCI	6	2/1/3	Duration, occurrence, coverage	Piton: dur −0.22 [−0.67, 0.23] ns · Das: dur 0.03 [−0.10, 0.16] ns, occ −0.20 [−0.46, 0.06] ns
C	AD	6	2/0/4	Duration, occurrence, coverage	Piton: occ −0.21 [−0.39, −0.04] * · Das: dur −0.02 [−0.32, 0.28] ns, occ −0.20 [−0.53, 0.14] ns discordant for occurrence
D	SCD	0	0/0/0	Duration, occurrence, coverage	Not estimated
D	MCI	4	0/1/3	Duration, occurrence, coverage	Piton: dur −0.31 [−0.50, −0.11] *, cov −0.32 [−0.45, −0.18] *, occ −0.19 † · Das: dur −0.26 [−0.48, −0.04] *, occ −0.11 [−0.34, 0.13] ns discordant for occurrence
D	AD	7	0/2/5	Duration, occurrence, coverage	Piton: occ −0.30 [−0.46, −0.15] * · Das: dur −0.16 [−0.36, 0.05] ns, occ −0.27 [−0.63, 0.10] ns discordant for occurrence

Abbreviations: ↑ = increase relative to HC; ↓ = decrease relative to HC. HC = healthy controls; SCD = subjective cognitive decline; MCI = mild cognitive impairment; AD = Alzheimer’s disease dementia. Data are presented as pooled effect estimates with 95% confidence intervals in square brackets. * Statistically significant (*p* < 0.05). † The 95% confidence interval was not reported in the source meta-analysis.

## Data Availability

All data extracted from the included studies and analyzed in this review are presented within the article and its Supplementary Materials (Supplementary Tables S1–S9). No new primary data were generated, and no custom analytic code was written for this review.

## Supplementary Materials

The following supporting information can be downloaded at: https://www.mdpi.com/article/10.3390/diagnostics16183019/s1, The Supplementary Materials comprise nine tables containing the study-level data, quality appraisal, search documentation and verification records underlying the synthesis reported in the article: Table S1: Characteristics of the studies included in the qualitative synthesis (*n* = 26 primary studies; two meta-analyses listed separately as contextual references); Table S2: Methodological quality assessment of the 26 included primary studies using the Newcastle–Ottawa Scale adapted for cross-sectional studies (NOS-CS), reported at domain level; Table S3: Complete database-specific search strategies; Table S4: AMSTAR 2 appraisal of the two contextual meta-analyses; Table S5: Per-outcome assessment of the feasibility of independent quantitative pooling; Table S6: Citation matrix quantifying overlap in primary studies between the two contextual meta-analyses and the present review; Table S7: Sensitivity analysis of the eligibility of each included study under the original a priori list of diagnostic frameworks (NIA-AA 2011/2018, DSM-5, ICD-10/11 only), before the amendment described in Section 2.1; Table S8: Analytical parameters bearing on cross-study commensurability, per included study; Table S9: Cell-by-cell attribution of the direction assignments in Table 1, with the verification status of each assignment. All references cited in the Supplementary Materials are included in the reference list of the main article.

## Abbreviations

The following abbreviations are used in this manuscript:

AAHC	Atomize and agglomerate hierarchical clustering
AD	Alzheimer’s disease
AMSTAR 2	A MeaSurement Tool to Assess systematic Reviews, version 2
APOE	Apolipoprotein E
AT(N)	Amyloid, tau, neurodegeneration
CCA	Corrected covered area
CDR	Clinical Dementia Rating
CI	Confidence interval
CSF	Cerebrospinal fluid
DMN	Default mode network
DSM-5	Diagnostic and Statistical Manual of Mental Disorders, 5th edition
EEG	Electroencephalography
EEGLAB	EEG Laboratory (MATLAB toolbox)
EMBASE	Excerpta Medica Database
fMRI	Functional magnetic resonance imaging
GRADE	Grading of Recommendations, Assessment, Development and Evaluations
HC	Healthy control
ICD-10	International Classification of Diseases, 10th revision
ICTRP	International Clinical Trials Registry Platform
IEEE	Institute of Electrical and Electronics Engineers
IWG-II	International Working Group, second iteration
MCI	Mild cognitive impairment
MEDLINE	Medical Literature Analysis and Retrieval System Online
MeSH	Medical Subject Headings
MICROSTATELAB	EEGLAB toolbox for resting-state microstate analysis
MMSE	Mini-Mental State Examination
MoCA	Montreal Cognitive Assessment
MRI	Magnetic resonance imaging
NIA-AA	National Institute on Aging–Alzheimer’s Association
NINCDS-ADRDA	National Institute of Neurological and Communicative Disorders and Stroke–Alzheimer’s Disease and Related Disorders Association
NOS-CS	Newcastle–Ottawa Scale adapted for cross-sectional studies
NR	Not reported
PET	Positron-emission tomography
PICO	Population, index test, comparator, outcome
PRISMA	Preferred Reporting Items for Systematic Reviews and Meta-Analyses
PROSPERO	International Prospective Register of Systematic Reviews
PsycARTICLES	American Psychological Association full-text journal collection
PsycINFO	Psychological Information Database
SCD	Subjective cognitive decline
SD	Standard deviation
SMD	Standardized mean difference
SWiM	Synthesis Without Meta-Analysis
TAAHC	Topographic atomize and agglomerate hierarchical clustering
WHO	World Health Organization
