# Supplementary material for: Resting-State EEG Microstate Dynamics as Neurophysiological Biomarkers Across the Alzheimer’s Disease Continuum: A Systematic Review
Source: Diagnostics (Basel). 2026 Sep 17;16(18):3019. doi: 10.3390/diagnostics16183019 (PMC13605323; doi:10.3390/diagnostics16183019)
Supplement: Supplementary file 1 [file diagnostics-16-03019-s001.zip › Supplementary.Materials.v5.pdf]

Supplementary Materials

Resting-State EEG Microstate Dynamics as Neurophysiological Biomarkers across the Alzheimer's Disease Continuum: A Systematic Review

Chanda Simfukwe, Seong Soo A. An, Young Chul Youn

Contents

Table S1. Characteristics of included studies and reasons for inclusion

Table S2. Methodological quality assessment (NOS-CS), domain level

Table S3. Full database-specific search strategies

Table S4. AMSTAR 2 appraisal of the two contextual meta-analyses

Table S5. Per-outcome assessment of pooling feasibility

Table S6. Citation matrix quantifying overlap between the two meta-analyses

Table S7. Sensitivity analysis: study eligibility under the original a priori diagnostic-framework criteria

Table S8. Analytical parameters bearing on cross-study commensurability

Table S9. Cell-by-cell attribution of the direction assignments in Table 1

Table S1. Characteristics of the studies included in the qualitative synthesis (n = 26 primary studies; two meta-analyses listed separately as contextual references).

#	Author, Year (Ref)	Country	Study Design	Total N	N per group (HC/SCD/MCI/AD/other)	Diagnostic Criteria	EEG Microstate Algorithm	MS Classes	Parameters Reported	Key Finding vs HC	ATN Biomarker Data	PMID	DOI	Reason for Inclusion (PICO)	Cognitive severity (mean per group, SD in parentheses)
SECTION A - Meta-Analyses (n = 2)															
A1	Das et al., 2026 [30]	Canada (multicentre)	Systematic review & meta-analysis	16 primary studies pooled (pooled N not stated)	HC, MCI, AD pooled across studies	Various (NIA-AA, DSM-IV/5)	Various (K-means majority)	4	Duration, Occurrence, Coverage	MS-A duration ↑ AD (g=0.41); MS-B duration ↑ AD (g=0.48); MS-D duration ↓ MCI (g=−0.26); MS-A occurrence ↑ MCI (g=0.40)	No	41896041	10.1016/j.clinph.2026.2111856	Contextual reference only appraised with AMSTAR 2 (Supplementary Table S4); not counted as an included primary study (see Section 2.7).	
A2	Piton et al., 2026 [31]	Switzerland / Sweden	Meta-analysis	Pooled N=1,347 (12 studies)	HC=385 / MCI=448 / AD=514	Various	Various (K-means majority)	4	Duration, Occurrence, Coverage	MS-A duration ↑ AD (g=0.440) and MCI (g=0.398); occurrence ↑ MCI (g=0.369); MS-B duration ↑ AD (g=0.399); MS-C occurrence ↓ AD (g=−0.213); MS-D all params ↓ MCI; occurrence ↓ AD (g=−0.304)	No	41496382	10.1016/j.nicl.2025.103929	Contextual reference only appraised with AMSTAR 2 (Supplementary Table S4); not counted as an included primary study (see Section 2.7).	
SECTION B Primary Studies (n = 26): ordered chronologically by publication year															
B1	Strik et al., 1997 [53] ‡	Germany	Cross-sectional	154	HC=21 / Subj.MI=31 / Obj.MI=38 / DAT=64	NINCDS-ADRDA (pre-NIA-AA)	Modified K-means (Lehmann)	4	Duration, Occurrence, Coverage	↓ MS duration and anteriorisation in mild/moderate DAT; severity-dependent gradient; correlates with cognitive decline	No	9437775	10.1016/S0925-4927(97)00054-1	P: Alzheimer-type dementia (NINCDS-ADRDA); C: healthy elderly controls; I: modified K-means microstates; O: duration, occurrence, coverage. Companion cohort with B2.	HC 29.2 [28-30] / AD(DAT) 19.5 [14-24] [MMSE; source: Das T1]
B2	Dierks et al., 1997 [52] ‡	Germany	Cross-sectional	154	HC=21 / Subj.MI=31 / Obj.MI=38 / DAT=64	NINCDS-ADRDA (pre-NIA-AA)	Modified K-means (Lehmann)	4	Duration, Occurrence, Coverage	Reduced MS durations and anterior shift in DAT; graded severity pattern	No	9295180	10.1007/BF01277666	P/C/I/O as B1 (same N=154 cohort; distinct microstate analysis). All PICO criteria met; companion paper to B1.	HC 29.0 (1.1) / SMI 28.8 (1.3) / OMI 27.2 (2.1) / mild DAT 23.2 (2.5) / mod DAT 15.3 (3.0) [MMSE; source: Das T1]
B3	Stevens & Kircher, 1998 [54]	Germany / Switzerland	Cross-sectional	96	Demented/impaired=31 / Healthy elderly=30 / Young controls=35	SIDAM	Adaptive segmentation (Lehmann)	NR	Duration, Number of microstates, EEG reactivity	↑ ultra-short MS, ↓ average MS duration in cognitively impaired/demented vs HC; EEG temporo-spatial changes differentiate cognitive decline	No	9840373	10.1007/s004060050047	P: Alzheimer-type/cognitively impaired group (SIDAM, a validated dementia instrument); C: healthy elderly controls; I: adaptive microstate segmentation; O: microstate duration reported.	NR
B4	Nishida et al., 2013 [50]	Japan / Switzerland	Cross-sectional	94	HC-old=19 / HC-young=18 / FTD=18 / SCZ=20 / AD=19	DSM-IV	Modified K-means	4	Duration, Occurrence, Coverage	No significant AD-vs-HC difference for any class or parameter (duration p = 0.350 [A],	No	23403263	10.1016/j.clinph.2013.01.005	P: dedicated AD subgroup (DSM-IV) within a mixed sample; C: age-matched older	HC 28.74 (1.91) / AD 21.05 (2.44)

#	Author, Year (Ref)	Country	Study Design	Total N	N per group (HC/SCD/MCI/AD/other)	Diagnostic Criteria	EEG Microstate Algorithm	MS Classes	Parameters Reported	Key Finding vs HC	ATN Biomarker Data	PMID	DOI	Reason for Inclusion (PICO)	Cognitive severity (mean per group, SD in parentheses)
										0.247 [B], 0.398 [C], 0.336 [D]; occurrence p = 0.643/0.428/0.818/0.267). The MS-C duration reduction reported by this study is FTD vs controls (p = 0.028), not AD vs controls. MS-D reduction is in schizophrenia.				HC; I: modified K-means; O: duration, occurrence, coverage. Mixed-syndrome carve-out met (AD analysed separately vs HC).	[MMSE; source: Das T1, Piton T2]
B5	Schumacher et al., 2020 [49]	United Kingdom (Newcastle)	Cross-sectional	87	HC=18 / AD=27 / LBD=42 (25 DLB + 17 PDD)	McKhann et al. 2011 (AD); McKeith et al. 2005 (DLB); Emre et al. 2007 (PDD)	TAAHC (topographic atomize & agglomerate hierarchical clustering)	5	Duration, Occurrence, Coverage, Transition probability	MS duration ↑ in LBD for ALL classes vs AD and HC (p<0.001); MS dynamics in AD largely comparable to HC (null finding for AD vs HC); MS duration correlates with cognitive fluctuation severity in LBD (ρ=0.56)	No	30938426	10.1093/brain/awz069	P: dedicated AD subgroup (McKhann 2011) within an LBD-primary study; C: HC; I: TAAHC; O: duration, occurrence, coverage, transition probability. AD analysed separately vs HC.	HC 29.2 (0.9) / AD 20.7 (4.3) [MMSE; source: Das T1, Piton T2]
B6	Musaeus et al., 2019 [46]	Denmark (two memory clinics)	Cross-sectional	123	HC=38 / MCI=27 / AD=17 recruited (37 / 25 / 15 analysed)	NIA-AA 2011	Modified K-means	4	Duration, Occurrence, Coverage	MS-A occurrence ↑ (p = 0.028) and coverage ↑ (p = 0.010) across groups; post hoc MCI vs HC p = 0.0411 and 0.0077, AD vs HC p = 0.0395 and 0.0066. MS-B, MS-C and MS-D not significant at either stage (all p ≥ 0.082). The study reports no significant correlation between microstate A parameters and MMSE, ACE, amyloid, total tau or phosphorylated tau.	No	31263397	10.3389/fnins.2019.00563	P: MCI and AD (NIA-AA 2011); C: HC; I: modified K-means; O: duration, occurrence, coverage. Full PICO met.	HC 29.1 (1.0) / MCI 27.6 (1.5) / AD 26.3 (3.2) [MMSE; source: Das T1, Piton T1-2]
B7	Smailovic et al., 2019 [35]	Sweden (multicentre memory clinics)	Cross-sectional	945	HC=308 / SCD=210 / MCI=230 / AD=197	NIA-AA 2011	Modified K-means	4	Duration, Occurrence, Coverage	Progressive gradient ↑ asymmetrical maps (A,B) / ↓ symmetrical maps (C,D) across HC→SCD→MCI→AD; MS-C ~ CSF Aβ42; MS-B ~ CSF p-tau	Yes CSF Aβ42, p-tau	31795039	10.1016/j.nicl.2019.102046	P: SCD, MCI and AD (NIA-AA 2011), CSF-validated; C: HC; I: modified K-means; O: duration, occurrence, coverage. Key SCD-stage evidence.	HC 29.3 (0.8) / MCI 27.3 (2.1) / AD 23.0 (4.3) [MMSE; source: Das T1]
B8	Tait et al., 2020 [48]	United Kingdom (Exeter + San Marino)	Cross-sectional + 4-year longitudinal follow-up	Training: HC=26 / AD=21 / MCI=25; Test cohort: HC=7 / AD=9	HC=26, AD=21, MCI stable=7, MCI converter=4 (SWE); HC=7, AD=9 (RSM)	DSM-IV + NINCDS-ADRDA (training); IWG-2 (test cohort)	Modified K-means (k-means++ init; Krzanowski-Lai criterion)	4	Duration, Lempel-Ziv complexity (syntax entropy), Coverage	MS transition entropy (LZC) ↓ in AD; combined with spectral slowing: sensitivity/specificity >80%; MCI-to-AD conversion classification 90.9% (n=11 preliminary)	No	33077823	10.1038/s41598-020-74790-7	P: AD and MCI (DSM-IV/NINCDS-ADRDA/IWG-2); C: HC; I: modified K-means; O: coverage, Lempel-Ziv (syntax) entropy. Two independent cohorts.	HC 29 (1.0) / MCI 25 (1.0) / AD 23 (3.0) [MMSE; source: Das T1]
B9	Musaeus et al., 2020 [33]	Denmark / Norway / Sweden / Finland / Germany / Switzerland (6 centres)	Cross-sectional	369	HC=135 / MCI=117 / AD=117	NIA-AA 2011	Modified K-means	4	Duration, Occurrence, Coverage	MS-A ↑ duration/occurrence/coverage in AD and MCI; left temporal MS changes predict cognitive decline; classification HC/AD=69.8%, HC/MCI=58.7%	No	32338460	10.1002/brb3.1630	P: MCI and AD (NIA-AA 2011); C: HC; I: modified K-means; O: duration, occurrence, coverage. Full PICO met (multicentre).	HC 28.91 (1.34) / MCI 27.11 (2.16) / AD 23.52 (3.79) [MMSE; source: Das T1, Piton T1-2]
B10	Lassi et	Germany /	Cross-	122	HC=19 / SCD=57 /	NIA-AA 2018;	AAHC (atomise-	4	Duration,	MS-C duration/coverage	Yes CSF	3709443	10.1016/j.nicl.2023.103407	P: SCD and MCI (NIA-	HC 29.06

#	Author, Year (Ref)	Country	Study Design	Total N	N per group (HC/SCD/MCI/AD/other)	Diagnostic Criteria	EEG Microstate Algorithm	MS Classes	Parameters Reported	Key Finding vs HC	ATN Biomarker Data	PMID	DOI	Reason for Inclusion (PICO)	Cognitive severity (mean per group, SD in parentheses)
	al., 2023 [32]	Austria (multicentre)	sectional		MCI=46	SCD-Initiative criteria (Jessen 2014)	and-agglomerate hierarchical clustering)		Occurrence, Coverage, Transition probability	↓ graded SCD→MCI vs HC (earliest detectable change at SCD); MS-C topography differs by CSF AD-like profile; MS-C outperforms spectral EEG in discriminating SCD from HC	Aβ42, p-tau (subset)	7		AA 2018/SCD-I), CSF subset; C: HC; I: AAHC; O: duration, occurrence, coverage, transition probability.	(1.13) / MCI 26.82 (2.23) [MMSE; source: Das T1, Piton T1]
B11	Lin et al., 2021 [55]	China (Peking Union Medical College Hospital, Beijing)	Cross-sectional	77	EOAD=30 / LOAD=9 / FTD=14 / HC=24 (HC≤65: n=18; HC>65: n=6)	IWG-II criteria (AD); Neary/McKhann 2001 (FTD)	K-means clustering (EEGLAB Microstate 1.1)	4	Duration, Occurrence, GFP peaks/sec, Transition probability	MS-B duration ↑ in EOAD; MS-B coverage negatively correlated with CSF Aβ42 (r=−0.833, p=0.010); stronger microstate abnormalities in EOAD than LOAD	Yes CSF Aβ42, p-tau, t-tau	34421518	10.3389/fnins.2021.687053	P: dedicated AD subgroup EOAD/LOAD (IWG-II) vs HC; C: HC; I: K-means; O: duration, occurrence, GFP peaks, transition probability. FTD analysed separately.	AD 12 (NR) / HC NR [MoCA; source: Das T1]
B12	Lian et al., 2021 [47]	China	Cross-sectional	132	HC=43 / MCI=46 / AD=43	NIA-AA 2011	AAHC	4	Duration, Occurrence, Coverage, Transition probability	MS-B duration ↑ (p = 0.008) and coverage ↑ (p = 0.007) in AD vs HC; MCI vs HC not significant for MS-B (both p = 1.00). MS-C coverage differs MCI vs AD (p = 0.017) but not AD vs HC (p = 0.25). MS-A and MS-D not significant.	No	34600244	10.1016/j.clinph.2021.08.015	P: MCI and AD (NIA-AA 2011); C: HC; I: modified K-means; O: duration, occurrence, coverage, transition probability.	HC 27.5 (1.5) / MCI 24.3 (3.3) / AD 18.6 (6.6) [MMSE; source: Das T1, Piton T1-2]
B13	Teipel et al., 2021 [56]	Germany (Rostock University Medical Center)	Cross-sectional (simultaneous EEG-fMRI)	28	AD=14 / HC=14 (age- and sex-matched)	NINCDS-ADRDA + NIA-AA 2011 (probable AD)	Modified K-means (EEGLAB Microstate toolbox)	4 (selected by cross-validation)	Duration, Occurrence, Coverage (MS topologies vs fMRI networks)	Microstates are labelled 1–4 with no canonical A–D assignment. Occurrence of microstate 2 ↑ and microstate 3 ↓ in AD vs HC (p = 0.02 each); no significant duration difference. The increase in frontal network expression is a resting-state fMRI component, not a microstate parameter. Withdrawn from the class-level counts in Table 1 (Supplementary Table S9).	No	34220668	10.3389/fneur.2021.637542	P: probable AD (NINCDS-ADRDA/NIA-AA 2011); C: age- and sex-matched HC; I: modified K-means; O: duration, occurrence, coverage (simultaneous EEG-fMRI).	HC 28.7 (0.8) / AD 24.6 (3.1) [MMSE; source: Das T1, Piton T2]
B14	Yan et al., 2024 [34]	China	Cross-sectional	94	HC=38 / AD=56	NIA-AA 2011	Modified K-means	4	Duration, Occurrence, Coverage	After FDR correction only MS-C duration differs significantly from HC (AD 0.069 vs HC 0.063, p = 0.016, p_FDR = 0.040). MS-D duration (p = 0.014, p_FDR = 0.070) and MS-B occurrence (p = 0.015, p_FDR = 0.075) do not survive correction; MS-A and all coverage parameters are non-significant. MS-B duration/occurrence ~ CSF Aβ1-42 and MS-C duration ~ CSF Aβ1-40 (p < 0.05); MS changes associate with Stroop and ADL scores.	Yes CSF Aβ1-42, Aβ1-40, p-tau, total-tau, NfL	38727873	10.1007/s11357-024-01181-5	P: AD (NIA-AA 2011), multi-biomarker CSF panel; C: HC; I: modified K-means; O: duration, occurrence, coverage.	HC 28.46 (1.40) / AD 18.72 (6.36) [MMSE; source: Das T1, Piton T2]
B15	Bejia et al., 2023	France (Université	Cross-sectional	NR	AD-with-vascular / AD-without-vascular /	NR	NR	NR	Duration, Occurrence	EEG MS alongside spectral and connectivity	No	37433259	10.1016/j.neurobiolaging.2023.06.008	P: AD subgroups within a memory-clinic sample;	AD+V 19.83

#	Author, Year (Ref)	Country	Study Design	Total N	N per group (HC/SCD/MCI/AD/other)	Diagnostic Criteria	EEG Microstate Algorithm	MS Classes	Parameters Reported	Key Finding vs HC	ATN Biomarker Data	PMID	DOI	Reason for Inclusion (PICO)	Cognitive severity (mean per group, SD in parentheses)
	[57]	de Lille / INSERM U1172)			Lewy body / VaD / HC				, Coverage (+ spectral / connectivity measures)	measures in multiple dementia types; VaD shows distinct pattern; AD-specific subgroup shows expected MS pattern vs HC				C: HC; O: duration, occurrence, coverage reported. Note: formal diagnostic criteria and per-group N not stated in the source publication.	(5.87) / AD-V 17.3 (8.4) / HC NR [MMSE; source: Das T1]
B16	Li Z et al., 2024 [58]	China (Yanshan University / Qinhuangdao Hospital)	Cross-sectional	NR	NR	NIA-AA (AD); MCI criteria not reported	NR	NR	Duration, Occurrence, Brain homeostasis coefficient (Bhc)	MS-B parameters ↑ in MCI vs HC; Bhc ↓ in MCI and AD, strongest in right frontal lobe; Bhc positively correlates with MoCA	No	38383723	10.1093/cercor/bhae043	P: AD (NIA-AA); C: HC; I: microstate segmentation; O: duration, occurrence reported vs HC. Note: MCI diagnostic criteria and per-group N not stated in the source.	MCI 25.90 (2.35) / AD 18.48 (5.50) / HC NR [MoCA; source: Das T1]
B17	Nobukawa et al., 2024 [59]	Japan (Chiba Institute of Technology; Kanazawa University)	Cross-sectional	32	HC=16 / AD=16 (no MCI group)	NINCDS-ADRDA + DSM-IV	Instantaneous-phase microstate analysis (novel method)	4	Instantaneous phase-based microstate state transitions	AD-specific state transitions among 4 states (defined by leading phase location) detected; phase dynamics disrupted beyond classical temporal parameter changes	No	38167950	10.1038/s41598-023-50265-3	P: AD (NINCDS-ADRDA/DSM-IV); C: HC; I: instantaneous-phase microstate analysis; O: microstate state-transition parameters (transition-probability outcome).	AD 15.5 (4.78) / HC NR [MMSE; source: Das T1]
B18	Wan et al., 2024 [60] †	China (Southeast University, Nanjing)	Cross-sectional	88	HC=29 / FTD=23 / AD=36	DSM-IIIR/IV + NINCDS-ADRDA	CEEMD-enhanced modified K-means	4	Microstate sequence non-randomness index (MSNRI)	MSNRI significantly lower in AD vs HC ($p<0.001$) and FTD vs HC ($p<0.01$); CEEMD-enhanced MSNRI predicts MMSE ($R^2=0.940$)	No	38790465	10.3390/brainsci14050487	P: dedicated AD subgroup (DSM/NINCDS-ADRDA) vs HC; C: HC; I: CEEMD-modified K-means; O: microstate sequence non-randomness index (sequence/syntax outcome).	NR
B19	Yang et al., 2024 [61] †	China (Henan University of Science and Technology, Luoyang)	Cross-sectional (OpenNeuro public dataset)	65	AD=36 / HC=29	NIA-AA (OpenNeuro dataset clinical labels)	NR	NR	Alpha-band MS features (Duration, Occurrence, Coverage + spectral comparisons)	SVM classifier using alpha-band MS features distinguishes AD from HC with 99.22% accuracy; alpha sub-band microstate feature set outperforms conventional EEG features across 4 classifiers (avg 98.61% vs 91.19%)	No	39666689	10.1371/journal.pone.0311958	P: AD vs HC (NIA-AA labels); C: HC; I: microstate segmentation; O: alpha-band microstate duration/occurrence/coverage. Note: parameters reported as classifier features rather than group-level comparisons.	NR
B20	Wu et al., 2025 [64]	China (Chengde Medical University, Hebei)	Cross-sectional	69	MCI=? / HC=? (total n=69; exact split not in abstract)	MCI criteria Petersen/NIA-AA	Modified K-means (SVM classifier)	4	Duration, Coverage, Entropy measures (PCA applied)	MCI showed ↑ coverage/duration in MS-C but ↓ in A, B, D vs HC; PCA revealed 2 principal components explaining >75% variance; ML models achieved high accuracy distinguishing MCI from HC	No	39772750	10.1177/13872877241305961	P: MCI (Petersen/NIA-AA); C: HC; I: modified K-means; O: duration, coverage reported vs HC. Note: per-group N not stated in the abstract.	MCI 18.46 (3.92) / HC 22.86 (4.50) [MoCA; source: Das T1]
B21	Tarailis et al., 2025 [63]	Switzerland (University of Geneva)	Cross-sectional	150	MCI=30 / Healthy older HC=60 / Healthy younger HC=60	MoCA-based MCI screening; formal criteria not reported	High-density EEG (hdEEG) microstate analysis (method not specified in abstract)	4 (A-D canonical)	Duration, Occurrence, Coverage; mind-wandering ARSQ correlates	MS-A ↑ in MCI vs age-matched healthy older controls (GEV $p = 0.011$, duration $p = 0.033$, coverage $p = 0.015$, occurrence $p = 0.031$). The MS-C reduction is against the younger control group; no significant MCI vs age-matched older contrast for MS-C is reported.	No	40210901	10.1038/s41598-025-97240-8	P: MCI vs older/younger HC; C: HC; I: high-density EEG microstates; O: duration, occurrence, coverage. Note: MCI defined by MoCA screening; formal diagnostic criteria not stated.	MCI 26.4 (3.3) / HC NR [MoCA; source: Das T1]

#	Author, Year (Ref)	Country	Study Design	Total N	N per group (HC/SCD/MCI/AD/other)	Diagnostic Criteria	EEG Microstate Algorithm	MS Classes	Parameters Reported	Key Finding vs HC	ATN Biomarker Data	PMID	DOI	Reason for Inclusion (PICO)	Cognitive severity (mean per group, SD in parentheses)
B22	Johansson et al., 2025 [62]	Sweden (Karolinska Institutet, Stockholm)	Cross-sectional	48	Non-carrier HC=24 / Pre-manifest carriers=17 / Symptomatic carriers=7 (total EEG recordings=99)	Monogenic AD mutation status (APP/PSEN1/PSEN2)	NR	7 (classes A-G)	Coverage (per class)	Seven-class solution. Coverage of class B ↓ and class C ↓ in symptomatic carriers vs non-carriers; coverage of class D ↓ and class E ↑ in all carriers. The report states its class B correlates only poorly to moderately with four-class class B maps. Withdrawn from the class-level counts in Table 1 (Supplementary Table S9).	No (genetic mutation status - not ATN)	41005266	10.1016/j.nicl.2025.103879	P: autosomal-dominant AD mutation carriers (APP/PSEN1/PSEN2) — genetically defined AD spectrum; C: non-carrier HC; I: microstate analysis; O: per-class coverage.	NR
B23	Chang, 2025 [65] †	USA (Williams College) / OpenNeuro	Cross-sectional	88	HC=29 / AD=36 / FTD=23	NINCDS-ADRDA; DSM-IIIR/IV; ICD-10	Modified K-means (MICROSTATELAB)	4 and 7	Duration, Occurrence, Coverage; ratio-normalised	Four-class solution: MS-B duration/coverage ↑ and MS-D occurrence ↓ in AD vs HC ($p < 0.001$); MS-A and MS-C not significant. Seven-class solution: MS-C ↓ and MS-G ↑ in AD vs HC — these are seven-class labels and do not map onto the four-class classes.	No	—	10.3390/medicina61111917	P: dedicated AD subgroup (NINCDS-ADRDA) vs HC; C: healthy controls; I: MICROSTATELAB modified K-means; O: duration, occurrence, coverage and ratio-normalised forms. Shared OpenNeuro dataset (see †).	HC 30.0 / AD 17.75 (4.5) / FTD 22.17 (8.22) [MMSE]
B24	Hou et al., 2025 [51] †	China (Tianjin) / OpenNeuro	Cross-sectional	65	HC=29 / AD=36	Per source dataset (NINCDS-ADRDA)	Modified K-means (4-class)	4	Coverage, Duration, Occurrence, Transition probabilities	21 of 24 microstate features differed significantly between AD and HC; SVM classification accuracy 75.8%	No	41004655	10.1177/13872877251379072	P: AD vs HC from a public dataset; C: healthy controls; I: microstate segmentation; O: group-level coverage, duration, occurrence and transition probabilities reported with between-group statistics. Shared OpenNeuro dataset (see †).	AD 17.75 (4.5) / HC NR [MMSE; source: Das T1]
B25	Lv et al., 2026 [66]	China (Shanghai) / BrainLat (Latin America)	Cross-sectional	123	HC=32 / AD=35 / bvFTD=18 / PD=16 / MS=22	NINCDS-ADRDA	Modified K-means (Microstate Analysis Toolbox)	4	Duration, Occurrence, Coverage	Post hoc HC vs AD (Bonferroni): MS-C duration ↑ ($p = 0.002$), occurrence ↑ ($p < 0.001$), coverage ↑ ($p < 0.001$); MS-D occurrence ↓ ($p = 0.028$) and coverage ↓ ($p = 0.018$). No significant HC-vs-AD contrast for MS-A or MS-B.	No	—	10.1155/ane/7974554	P: dedicated AD subgroup (NINCDS-ADRDA) within a cross-disease sample; C: age-matched HC; I: modified K-means; O: duration, occurrence, coverage with post-hoc HC-vs-AD contrasts. Shared BrainLat cohort.	HC 25.52 (3.18) / AD 17.03 (3.41) [MoCA]
B26	Sayman et al., 2026 [67]	Türkiye (Alanya / Istanbul)	Cross-sectional	60	HC=30 / MCI=30	Petersen criteria; DSM-5 Mild Neurocognitive Disorder	K-means (MICROSTATELAB; Metamaps2023)	7 (classes A-G)	GEV, Duration, Occurrence, Coverage	MCI vs HC: GEV of MS-B ($p=0.007$) and MS-C ($p=0.007$) ↑; occurrence and coverage of B and C ↑; duration unchanged	No	—	10.3389/fnagi.2026.1734828	P: MCI by Petersen/DSM-5 criteria; C: age- and sex-matched HC; I: MICROSTATELAB seven-class solution; O: GEV plus duration, occurrence and coverage with group comparisons.	HC 28.90 (1.24) / MCI 25.07 (2.60) [MMSE]

Per-group cognitive scores were extracted from the participant-demographic tables of the two contextual meta-analyses [30,31] (Das et al., Table 1; Piton et al., Tables 1–2), which tabulate these values from the primary studies. Twenty-two of the 26 included studies reported a global cognitive measure (17 MMSE, 5 MoCA); four reported neither. These counts correspond to Section 3.2.1 of the manuscript.

Abbreviations: HC=healthy controls; SCD=subjective cognitive decline; MCI=mild cognitive impairment; AD=Alzheimer's disease dementia; FTD=frontotemporal dementia; LBD=Lewy body dementia; DLB=dementia with Lewy bodies; PDD=Parkinson's disease dementia; VaD=vascular dementia; EOAD=early-onset AD; LOAD=late-onset AD; DAT=dementia of Alzheimer type; SCZ=schizophrenia; Subj.MI=subjective memory impairment; Obj.MI=objective memory impairment; MS=microstate; AAHC/TAAHC=(topographic) atomize-and-agglomerate hierarchical clustering; CEEMD=complementary ensemble empirical mode decomposition; NIA-AA=National Institute on Aging–Alzheimer's Association; IWG-II=International Working Group 2; ATN=amyloid/tau/neurodegeneration; CSF=cerebrospinal fluid; PLI=phase lag index; LZC=Lempel–Ziv complexity; Bhc=brain homeostasis coefficient; MSNRI=microstate sequence non-randomness index; MMSE=Mini-Mental State Examination; MoCA=Montreal Cognitive Assessment; SVM=support vector machine; GFP=global field power; NR=not reported in the source publication.

Bracketed numbers in the "Author, Year (Ref)" column are the corresponding reference numbers in the main manuscript reference list. Green cells indicate that ATN biomarker data were available.

† Studies marked with a dagger (B18 Wan 2024 [60], B19 Yang 2024 [61], B23 Chang 2025 [65], B24 Hou 2025 [51]) analyze the same publicly available OpenNeuro EEG dataset (AD=36, HC=29; Wan 2024 [60] and Chang 2025 [65] additionally include FTD=23 from the same source). They do not contribute independent participants and are grouped as a shared-dataset cluster in the narrative synthesis; this overlap is acknowledged as a limitation in Section 4.5 of the manuscript.

‡ Studies marked with a double dagger (B1 Strik 1997 [53] [, B2 Dierks 1997 [52]) are companion papers reporting different analyses of the same N=154 cohort; they are counted as two publications but represent one independent sample.

Three studies assessed at full text were excluded during re-screening against the eligibility criteria: one because no healthy-control comparator was reported, and two because no extractable microstate parameter was reported. Consistent with the treatment of the category-level exclusions reported in Section 3.1, these records are reported by reason rather than cited individually.

Table S2. Methodological quality assessment of the 26 included primary studies using the Newcastle-Ottawa Scale adapted for cross-sectional studies (NOS-CS), reported at domain level.

Study	Ref	S1 Representative sample	S2 Sample size ≥30/group	S3 Non-respon-dents	S4 Validated diagnostic criteria	S5 EEG outcome validated	Selection Total (/5)	C1 Age controlled	C2 Additional confounders	Comparability Total (/2)	O1 Assessment standardised	O2 Statistical test	O3 Analysts blinded	Outcome Total (/3)	NOS-CS Total (/10)	★ Stars	Quality Grade	Notes
Strik et al., 1997 ‡	[53]	★	★	☆	★	★	4/5	★	☆	1/2	★	★	☆	2/3	7/10	★★★★★★★☆☆☆	High (7–10★)	NINCDS-ADRDA; companion paper shares N=154 cohort with Dierks 1997; no blinding
Dierks et al., 1997 ‡	[52]	★	★	☆	★	★	4/5	★	☆	1/2	★	★	☆	2/3	7/10	★★★★★★★☆☆☆	High (7–10★)	NINCDS-ADRDA; companion paper shares N=154 cohort with Strik 1997; no blinding
Stevens & Kircher, 1998	[54]	☆	★	☆	★	★	3/5	★	☆	1/2	★	★	☆	2/3	6/10	★★★★★★☆☆☆☆	Moderate (4–6★)	SIDAM criteria; mixed cognitively impaired group; MS classes NR; no blinding
Nishida et al., 2013	[50]	★	☆	☆	★	★	3/5	★	★	2/2	★	★	☆	2/3	7/10	★★★★★★★☆☆☆	High (7–10★)	DSM-IV; small AD subgroup (n=19); HC-old matched; no blinding
Schumacher et al., 2020	[49]	☆	★	☆	★	★	3/5	★	☆	1/2	★	★	☆	2/3	6/10	★★★★★★☆☆☆☆	Moderate (4–6★)	McKhann 2011/McKeith 2005/Emre 2007; primary population LBD; AD null finding vs HC; TAAHC 5-class; no blinding
Musaeus et al., 2019	[46]	★	★	★	★	★	5/5	★	★	2/2	★	★	☆	2/3	9/10	★★★★★★★★★☆	High (7–10★)	NIA-AA 2011; multicentre 4 sites; N=123; no blinding
Smailovic et al., 2019	[35]	★	★	★	★	★	5/5	★	★	2/2	★	★	☆	2/3	9/10	★★★★★★★★★☆	High (7–10★)	NIA-AA 2011; large N=945; multicentre; CSF biomarker validation; no blinding
Tait et al., 2020	[48]	☆	★	☆	★	★	3/5	★	★	2/2	★	★	☆	2/3	7/10	★★★★★★★☆☆☆	High (7–10★)	DSM-IV + NINCDS-ADRDA + IWG-2; two independent cohorts; no blinding
Musaeus et al., 2020	[33]	★	★	★	★	★	5/5	★	★	2/2	★	★	☆	2/3	9/10	★★★★★★★★★☆	High (7–10★)	NIA-AA 2011; 6-centre N=369; no blinding
Lassi et al., 2023	[32]	★	★	★	★	★	5/5	★	★	2/2	★	★	☆	2/3	9/10	★★★★★★★★★☆	High (7–10★)	NIA-AA 2018 + SCD-I; multicentre; CSF subset; AAHC validated; no blinding
Lin et al., 2021	[55]	☆	★	☆	★	★	3/5	★	★	2/2	★	★	☆	2/3	7/10	★★★★★★★☆☆☆	High (7–10★)	IWG-II (AD)/Neary-McKhann (FTD); N=77; CSF biomarker validation; EOAD focus; no blinding
Lian et al., 2021	[47]	★	★	☆	★	★	4/5	★	★	2/2	★	★	☆	2/3	8/10	★★★★★★★★☆☆	High (7–10★)	NIA-AA 2011; China single-centre; N=132; no blinding
Teipel et al., 2021	[56]	☆	☆	☆	★	★	2/5	★	☆	1/2	★	★	☆	2/3	5/10	★★★★★☆☆☆☆☆	Moderate (4–6★)	NINCDS-ADRDA + NIA-AA 2011; small N=28 (14/14); simultaneous EEG-fMRI; no blinding
Yan et al., 2024	[34]	★	★	☆	★	★	4/5	★	★	2/2	★	★	☆	2/3	8/10	★★★★★★★★☆☆	High (7–10★)	NIA-AA (version NR 2011 or 2018 not specified in abstract); N=94; CSF multi-biomarker panel; no blinding
Bejia et al., 2023	[57]	☆	☆	☆	☆	★	1/5	★	☆	1/2	★	★	☆	2/3	4/10	★★★★☆☆☆☆☆☆	Moderate (4–6★)	France (Univ. Lille/INSERM U1172); mixed

Study	Ref	S1 Representative sample	S2 Sample size ≥30/group	S3 Non-respon-dents	S4 Validated diagnostic criteria	S5 EEG outcome validated	Selection Total (/5)	C1 Age controlled	C2 Additional confounders	Comparability Total (/2)	O1 Assessment standardised	O2 Statistical test	O3 Analysts blinded	Outcome Total (/3)	NOS-CS Total (/10)	★ Stars	Quality Grade	Notes
																		dementia types; N NR; criteria NR; AD subgroup present; no blinding | Sample-size star not awarded: per-group N not reported.
Li Z et al., 2024	[58]	☆	☆	☆	☆	★	1/5	★	☆	1/2	★	★	☆	2/3	4/10	★★★★☆☆☆☆☆☆	Moderate (4–6★)	China (Yanshan Univ.); N NR; MCI criteria NR; novel Bhc metric; no blinding | Sample-size star not awarded: per-group N not reported.
Nobukawa et al., 2024	[59]	☆	☆	☆	★	★	2/5	★	☆	1/2	★	★	☆	2/3	5/10	★★★★★☆☆☆☆☆	Moderate (4–6★)	NINCDS-ADRDA + DSM-IV; small N=32 (HC=16/AD=16); novel phase-based method; no blinding
Wan et al., 2024 †	[60]	☆	★	☆	★	★	3/5	★	☆	1/2	★	★	☆	2/3	6/10	★★★★★★☆☆☆☆	Moderate (4–6★)	DSM-IIIR/IV + NINCDS-ADRDA; N=88; OpenNeuro shared dataset — not independent; no blinding
Yang et al., 2024 †	[61]	☆	★	☆	★	★	3/5	★	☆	1/2	★	★	☆	2/3	6/10	★★★★★★☆☆☆☆	Moderate (4–6★)	NIA-AA (OpenNeuro); N=65; OpenNeuro shared dataset — not independent; alpha-band classifier; no blinding
Wu et al., 2025	[64]	☆	☆	☆	☆	★	1/5	★	☆	1/2	★	★	☆	2/3	4/10	★★★★☆☆☆☆☆☆	Moderate (4–6★)	China (Chengde); N=69 total (MCI/HC split not in abstract); criteria NR; ML classification; no blinding | Sample-size star not awarded: per-group N not reported.
Tarailis et al., 2025	[63]	☆	★	☆	☆	★	2/5	★	★	2/2	★	★	☆	2/3	6/10	★★★★★★☆☆☆☆	Moderate (4–6★)	MoCA-based screening (formal criteria NR); N=150; Switzerland; no blinding
Johansson et al., 2025	[62]	★	☆	☆	★	★	3/5	★	★	2/2	★	★	☆	2/3	7/10	★★★★★★★☆☆☆	High (7–10★)	Monogenic AD mutation status (APP/PSEN1/PSEN2); specialised genetic cohort; small N=48; 7-class; no blinding
Chang, 2025	[65]	★	★	☆	★	★	4/5	★	★	2/2	★	★	☆	2/3	8/10	★★★★★★★★☆☆	High (7–10★)	OpenNeuro dataset; NINCDS-ADRDA/DSM; ANCOVA with age and gender; Bonferroni correction; no analyst blinding
Hou et al., 2025	[51]	★	★	☆	☆	★	3/5	☆	☆	0/2	★	★	☆	2/3	5/10	★★★★★☆☆☆☆☆	Moderate (4–6★)	OpenNeuro dataset; diagnostic criteria not restated in report; no age/sex adjustment described; no blinding
Lv et al., 2026	[66]	★	★	☆	★	★	4/5	★	☆	1/2	★	★	☆	2/3	7/10	★★★★★★★☆☆☆	High (7–10★)	BrainLat cohort; NINCDS-ADRDA; ANCOVA with age as covariate;

Study	Ref	S1 Representative sample	S2 Sample size ≥30/group	S3 Non-respon-dents	S4 Validated diagnostic criteria	S5 EEG outcome validated	Selection Total (/5)	C1 Age controlled	C2 Additional confounders	Comparability Total (/2)	O1 Assessment standardised	O2 Statistical test	O3 Analysts blinded	Outcome Total (/3)	NOS-CS Total (/10)	★ Stars	Quality Grade	Notes
																		Bonferroni-corrected post hoc; no blinding
Sayman et al., 2026	[67]	★	★	☆	★	★	4/5	★	★	2/2	★	★	☆	2/3	8/10	★★★★★★★★☆☆	High (7–10★)	Petersen/DSM-5 criteria; age- and sex-matched groups; FDR correction; education adjusted; no blinding
QUALITY DISTRIBUTION SUMMARY																		
High quality (7–10★): n = 15 (57.7%)																		
Moderate quality (4–6★): n = 11 (42.3%)																		
Low quality (0–3★): n = 0 (0%)																		
Mean NOS-CS score: 6.69 / 10																		

Per-group cognitive scores were extracted from the participant-demographic tables of the two contextual meta-analyses [30,31] (Das et al., Table 1; Piton et al., Tables 1–2), which tabulate these values from the primary studies. Twenty-two of the 26 included studies reported a global cognitive measure (17 MMSE, 5 MoCA); four reported neither. These counts correspond to Section 3.2.1 of the manuscript.

Abbreviations: HC=healthy controls; SCD=subjective cognitive decline; MCI=mild cognitive impairment; AD=Alzheimer's disease dementia; FTD=frontotemporal dementia; LBD=Lewy body dementia; DLB=dementia with Lewy bodies; PDD=Parkinson's disease dementia; VaD=vascular dementia; EOAD=early-onset AD; LOAD=late-onset AD; DAT=dementia of Alzheimer type; SCZ=schizophrenia; Subj.MI=subjective memory impairment; Obj.MI=objective memory impairment; MS=microstate; AAHC/TAAHC=(topographic) atomize-and-agglomerate hierarchical clustering; CEEMD=complementary ensemble empirical mode decomposition; NIA-AA=National Institute on Aging–Alzheimer's Association; IWG-II=International Working Group 2; ATN=amyloid/tau/neurodegeneration; CSF=cerebrospinal fluid; PLI=phase lag index; LZC=Lempel–Ziv complexity; Bhc=brain homeostasis coefficient; MSNRI=microstate sequence non-randomness index; MMSE=Mini-Mental State Examination; MoCA=Montreal Cognitive Assessment; SVM=support vector machine; GFP=global field power; NR=not reported in the source publication.

Bracketed numbers in the "Author, Year (Ref)" column are the corresponding reference numbers in the main manuscript reference list. Green cells indicate that ATN biomarker data were available.

† Studies marked with a dagger (B18 Wan 2024 [60], B19 Yang 2024 [61], B23 Chang 2025 [65], B24 Hou 2025 [51]) analyze the same publicly available OpenNeuro EEG dataset (AD=36, HC=29; Wan 2024 and Chang 2025 additionally include FTD=23 from the same source). They do not contribute independent participants and are grouped as a shared-dataset cluster in the narrative synthesis; this overlap is acknowledged as a limitation in Section 4.5 of the manuscript.

‡ Studies marked with a double dagger (B1 Strik 1997 [53], B2 Dierks 1997 [52]) are companion papers reporting different analyses of the same N=154 cohort; they are counted as two publications but represent one independent sample.

Three studies assessed at full text were excluded during re-screening against the eligibility criteria: one because no healthy-control comparator was reported, and two because no extractable microstate parameter was reported. Consistent with the treatment of the category-level exclusions reported in Section 3.1, these records are reported by reason rather than cited individually.

Table S3. Complete database-specific search strategies.

Two bibliographic databases were searched from 1 January 1990 to 30 June 2026. Final search executed on 30 June 2026. No language restriction was applied at the search stage; English-language restriction was applied at full-text eligibility assessment.

A. PubMed / MEDLINE (searched via https://pubmed.ncbi.nlm.nih.gov)

Set	Query (as entered)	Field tag / vocabulary
#1	"Electroencephalography"[MeSH Terms] OR EEG[tiab] OR electroencephalogra *[tiab]	MeSH + title/abstract [tiab]
#2	Microstate *[tiab]	title/abstract [tiab]; truncation *
#3	"Alzheimer Disease"[MeSH Terms] OR "Cognitive Dysfunction"[MeSH Terms] OR Alzheimer *[tiab] OR dementia[tiab] OR "mild cognitive impairment"[tiab] OR MCI[tiab] OR "subjective cognitive decline"[tiab] OR SCD[tiab] OR "cognitive impairment"[tiab]	MeSH + title/abstract [tiab]
#4	#1 AND #2 AND #3	Boolean combination
Filters	Humans[Filter]; Publication date 1990/01/01:2026/06/30	Species + date limits

B. EMBASE (searched via Elsevier, https://www.embase.com)

Set	Query (as entered)	Field tag / vocabulary
#1	'electroencephalography'/exp OR 'electroencephalogram'/exp OR EEG:ti,ab OR electroencephalogra *:ti,ab	Emtree /exp + :ti,ab
#2	Microstate *:ti,ab	:ti,ab; truncation *
#3	'Alzheimer disease'/exp OR 'mild cognitive impairment'/exp OR 'cognitive defect'/exp OR Alzheimer*:ti,ab OR dementia:ti,ab OR 'mild cognitive impairment':ti,ab OR MCI:ti,ab OR 'subjective cognitive decline':ti,ab OR SCD:ti,ab	Emtree /exp + :ti,ab
#4	#1 AND #2 AND #3	Boolean combination
Limits	'human'/de AND [1990-2026]/py	Species + publication-year limits

C. Supplementary search procedures, deduplication, and yield

Hand-searching: Backward citation searching of the reference lists of all included studies and relevant systematic reviews/meta-analyses was performed manually by one reviewer and cross-checked by a second reviewer. Any additional records identified were subjected to the same two-stage screening process as records retrieved through the electronic searches.

Deduplication: Records retrieved from PubMed/MEDLINE and EMBASE were imported into Rayyan (Qatar Computing Research Institute, Doha, Qatar). Duplicate records were removed using Rayyan's automated deduplication function followed by manual verification.

Yield: The PubMed/MEDLINE and EMBASE searches retrieved 292 records. After removal of 101 duplicates, 191 unique records proceeded to title and abstract screening.

D. Web of Science Core Collection (searched via Clarivate)

Set	Query (as entered)	Field tag / vocabulary
#1	TS=(EEG OR electroencephalogra*)	TS = Topic (title, abstract, keywords, KeyWords Plus)
#2	TS=(microstate *)	TS; truncation *
#3	TS=(Alzheimer * OR dementia OR "mild cognitive impairment" OR MCI OR "subjective cognitive decline" OR SCD OR "cognitive impairment")	TS
#4	#1 AND #2 AND #3	Boolean combination
Limits	Timespan 1990-2026; Document types: Article, Review, Proceedings Paper	Date + document-type limits
Yield (Web of Science)	145 records; searched 5 August 2026	Executed as written

E. Scopus (searched via Elsevier)

Set	Query (as entered)	Field tag / vocabulary
#1	TITLE-ABS-KEY(EEG OR electroencephalogra	TITLE-ABS-KEY

Set	Query (as entered)	Field tag / vocabulary
	*)	
#2	TITLE-ABS-KEY(microstate *)	TITLE-ABS-KEY; truncation *
#3	TITLE-ABS-KEY(Alzheimer * OR dementia OR "mild cognitive impairment" OR MCI OR "subjective cognitive decline" OR SCD OR "cognitive impairment")	TITLE-ABS-KEY
#4	#1 AND #2 AND #3 AND PUBYEAR > 1989 AND PUBYEAR < 2027	Boolean + PUBYEAR limits
Yield (Scopus)	126 records (searched 5 August 2026)	Executed as written

F. PsycINFO (search strategy prepared but not executed)

Set	Query (as entered)	Field tag / vocabulary
#1	TI(EEG OR electroencephalogra *) OR AB(EEG OR electroencephalogra *) OR DE "Electroencephalography"	TI/AB free text + DE thesaurus descriptor
#2	TI(microstate*) OR AB(microstate *)	TI/AB; truncation *
#3	DE "Alzheimer's Disease" OR DE "Cognitive Impairment" OR TI(Alzheimer * OR dementia OR "mild cognitive impairment" OR MCI OR "subjective cognitive decline") OR AB(same terms)	DE thesaurus + TI/AB
#4	#1 AND #2 AND #3	Boolean combination
Limits	Publication year 1990-2026; Human population	Date + population limits
Yield (PsycINFO)	Not searched	PsycINFO was unavailable through the authors' institutional subscription; this is acknowledged as a limitation in Section 4.5.

G. IEEE Xplore

Set	Query (as entered)	Field tag / vocabulary
#1	("All Metadata":EEG OR "All Metadata":electroencephalogra *)	All Metadata field
#2	("All Metadata":microstate *)	All Metadata; truncation *
#3	("All Metadata":Alzheimer * OR "All Metadata":dementia OR "All Metadata":"mild cognitive impairment" OR "All Metadata":MCI)	All Metadata
#4	#1 AND #2 AND #3	Boolean combination
Limits	Year 1990-2026; Content types: Conferences, Journals	Date + content-type limits
Yield (IEEE Xplore)	9 records; searched 5 August 2026	Executed as written

H. Registration sources

Set	Query (as entered)	Field tag / vocabulary
PROSPERO	EEG microstate AND (Alzheimer OR dementia OR "cognitive impairment")	https://www.crd.york.ac.uk/prospero - to identify ongoing or completed systematic reviews
ClinicalTrials.gov	Condition: Alzheimer Disease OR Mild Cognitive Impairment; Other terms: EEG microstate	https://clinicaltrials.gov - to identify ongoing or unpublished trials
ICTRP (WHO)	EEG microstate AND Alzheimer	https://trialsearch.who.int
Yield (registries)	47 records; searched 5 August 2026: PROSPERO 24, ClinicalTrials.gov 10, WHO ICTRP 13	All were protocols or trial registrations; none eligible

I. Revised overall yield after database expansion

Item	Detail	Notes
Total records identified from all electronic sources	619	292 from PubMed/MEDLINE and EMBASE + 182 from Scopus, IEEE Xplore and the three registration sources + 145 from Web of Science Core Collection
Duplicate or previously identified records removed	160	101 from the initial search + 29 from the Scopus/IEEE Xplore/registration-source search + 30 Web of Science records already identified
Unique records screened at title/abstract	459	—
Records excluded at title/abstract	383	—
Full-text reports assessed for eligibility	76	—

Full-text reports excluded	48	Reasons shown in Figure 2 and Section 3.1
Primary studies included in the systematic review	26	Included in the narrative synthesis
Systematic reviews/meta-analyses retained as contextual references	2	Appraised using AMSTAR 2 and not counted as included primary studies

Sections D–H report the supplementary information-source searches conducted on 5 August 2026. These comprised Scopus, IEEE Xplore, Web of Science Core Collection, PROSPERO, ClinicalTrials.gov, and WHO ICTRP. PsycINFO was not searched because it was unavailable through the authors' institutional subscription; this limitation is acknowledged in Section 4.5. The prepared PsycINFO strategy is retained above for transparency and potential use in future updates of the review.

Table S4. AMSTAR 2 appraisal of the two contextual meta-analyses.

#	AMSTAR 2 item	Critical	Das et al. 2026	Piton et al. 2026	Basis for judgement
1	Research question and inclusion criteria included PICO components		Y	Y	Both state population, index measure, comparator and outcomes explicitly.
2	Protocol registered before commencement of the review	Critical	Y	N	Das: registered with PROSPERO (CRD42024565312). Piton: states explicitly "This study was not preregistered."
3	Explanation of selection of study designs for inclusion		Y	Y	Both restrict to resting-state EEG microstate studies with control groups and justify this.
4	Comprehensive literature search strategy	Critical	PY	PY	Das: PubMed, PsycINFO, EMBASE, MEDLINE; English and human filters; no grey literature or trial registries. Piton: searched PubMed, PsycINFO and Web of Science but retained only the PubMed results as the others were judged redundant.
5	Study selection performed in duplicate		Y	PY	Das: search and screening performed by two investigators (SD, BM). Piton: does not state that selection was duplicated.
6	Data extraction performed in duplicate		PY	PY	Neither publication states explicitly that extraction was performed independently in duplicate.
7	List of excluded studies with justifications	Critical	PY	N	Das: gives reasons for excluding the four pre-2000 studies and reports category counts. Piton: PRISMA diagram gives counts and broad reasons only; no list of excluded studies.
8	Included studies described in adequate detail		Y	Y	Both tabulate participant demographics, sample sizes, and methodological detail per study.
9	Satisfactory technique for assessing risk of bias in included studies	Critical	Y	N	Das: three modified Newcastle-Ottawa Scales applied to all 30 included studies (their Fig. 19). Piton: no formal risk-of-bias or quality appraisal is reported.
10	Sources of funding of included studies reported		N	N	Neither publication reports the funding sources of the individual included studies.
11	Appropriate meta-analytical (statistical) methods	Critical	Y	Y	Das: random-effects models, Hedges g, meta package. Piton: two-level mixed-effects model, Hedges g, metafor, inverse-variance weighting.
12	Assessment of the impact of risk of bias on results		Y	N	Das: sensitivity analyses excluding studies with estimated input data and studies at high risk of bias.

#	AMSTAR 2 item	Critical	Das et al. 2026	Piton et al. 2026	Basis for judgement
					Piton: no risk-of-bias assessment, so no such analysis.
13	Risk of bias accounted for when interpreting results	Critical	Y	N	Das: interprets pooled estimates in light of risk-of-bias ratings and heterogeneity. Piton: no risk-of-bias information is available to inform interpretation.
14	Satisfactory explanation of, and investigation into, heterogeneity		Y	PY	Das: reports I-squared for each outcome and investigates heterogeneity through sensitivity analyses. Piton: reports prediction intervals and discusses heterogeneity narratively, but does not report a heterogeneity statistic.
15	Investigation of publication bias (small-study effects)	Critical	Y	N	Das: funnel plots plus Egger regression test; reports significant asymmetry for AD vs HC (z = 3.10, p = 0.002). Piton: no assessment of publication bias.
16	Conflicts of interest and funding of the review reported		Y	Y	Both include declarations of competing interests and funding.
	Critical flaws (rated "No" on a critical domain)		0	5 (items 2, 7, 9, 13, 15)	
	Non-critical weaknesses		2 (items 6, 10)	3 (items 5, 6, 10)	
	OVERALL CONFIDENCE RATING		Moderate	Critically low	

Rating logic (Shea et al. 2017). High = no or one non-critical weakness; Moderate = more than one non-critical weakness; Low = one critical flaw; Critically low = more than one critical flaw. Das et al. has no critical flaw but more than one non-critical weakness, giving Moderate confidence. Piton et al. has five critical flaws, giving Critically low confidence; this reflects principally the absence of protocol registration, of any risk-of-bias appraisal of the included studies, and of any assessment of publication bias. Y = yes; PY = partial yes; N = no.

Item-level judgements were made against the full texts of Das et al. (2026) and Piton et al. (2026); the basis for each judgement is given in the right-hand column.

Table S5. Per-outcome assessment of the feasibility of independent quantitative pooling.

Outcome (parameter)	Comparison	Independent samples contributing (k) and publications reporting the outcome	Aggregate pooled estimate already published?	Summary statistics available for inverse-variance pooling	Specific barrier to independent pooling in this review
Duration (MS A–D)	MCI vs HC	k = 2–6 independent samples per microstate class (Table 1). Nine publications report duration with both an MCI and a healthy-control group: Musaeus 2019 [46], Musaeus 2020 [33], Tait 2020 [48], Lassi 2023 [32], Lian 2021 [47], Li Z 2024 [58], Wu 2025 [64], Tarailis 2025 [63], Sayman 2026 [67]. k is lower than nine because not every publication reports a contrast for every class.	Yes, Das 2026 [30] (MS-D duration g = −0.26); Piton 2026 [31] (MS-A duration g = 0.398; MS-D duration g = −0.306).	Not tabulable retrospectively. The extraction form recorded direction of effect and, where stated, the significance of each contrast; it did not systematically record group means with dispersion or a standardised effect estimate with its confidence interval (Sections 2.5, 4.5). Per-study availability of the statistics required for inverse-variance weighting therefore cannot be reported here without re-extraction from the primary reports.	Not a validity constraint. Random-effects estimates over a substantially overlapping set of these same primary studies are already published (CCA 57.7%; Table S6), so an independent pooling would add little (Section 2.7). Independently, class labels are not interchangeable across studies unless template maps are re-sorted to a common reference, which cannot be done post hoc from published summary statistics; and Wu 2025 [64] reports PCA-transformed rather than raw parameters.
Occurrence (MS A–D)	MCI vs HC	k = 2–6 per class (Table 1). Eight publications report occurrence with an MCI and a healthy-control group: Musaeus 2019 [46], Musaeus 2020 [33], Lassi 2023 [32], Lian 2021 [47], Li Z 2024 [58], Tarailis 2025 [63], Sayman 2026 [67], Wu 2025 [64] (as coverage/entropy).	Yes, Das 2026 [30] (MS-A occurrence g = 0.40); Piton 2026 [31] (MS-A occurrence g = 0.369; MS-D occurrence g = −0.190).	As above. In addition, Piton et al. (2026) [31] report an identical confidence interval, test statistic and p-value for the MS-D duration and occurrence analyses (Section 3.3.2), so the published occurrence interval is not usable as a benchmark.	As for duration: limited incremental value rather than invalidity, plus post hoc class-label non-equivalence. Sayman 2026 [67] reports global explained variance alongside occurrence, which is not the same quantity.
Coverage (MS A–D)	MCI vs HC	k = 2–6 per class (Table 1). Reported by Musaeus 2019 [46], Musaeus 2020 [33], Tait 2020 [48], Lassi 2023 [32], Lian 2021 [47], Wu 2025 [64], Tarailis 2025 [63], Sayman 2026 [67].	Yes, Piton 2026 [31] (MS-A coverage g = 0.462; MS-D coverage g = −0.319); Das 2026 [30] for selected classes.	As above.	As for duration. In addition, coverage is expressed on non-commensurable scales across the evidence base-absolute percentages in most reports, ratio-normalised values in Chang 2025 [65], and alpha-band-restricted values in Yang 2024 [61].
Duration (MS A–D)	AD vs HC	k = 4–6 per class (Table 1). Fourteen independent samples report duration with an AD and a healthy-control group, including the Strik/Dierks 1997 companion cohort [52, 53] and the OpenNeuro cluster [51, 60, 61, 65], each counted once.	Yes, Das 2026 [30] (MS-A duration g = 0.41; MS-B duration g = 0.48); Piton 2026 [31] (MS-A duration g = 0.440; MS-B duration g = 0.399).	As above.	Not a validity constraint; the same incremental-value argument applies (Section 2.7). Scale non-commensurability is more marked at this stage: Chang 2025 [65] reports ratio-normalised parameters, Yang 2024 [61] alpha-band-restricted parameters, and Li Z 2024 [58] a derived brain-homeostasis coefficient, none of which are on the same scale as the absolute values reported elsewhere.

Occurrence (MS A–D)	AD vs HC	k = 4–6 per class (Table 1); same contributing samples as for duration, less Tait 2020 [48], which does not report occurrence.	Yes, Piton 2026 [31] (MS-C occurrence g = −0.213; MS-D occurrence g = −0.304); Das 2026 [30] for selected classes.	As above.	As for duration at this stage.
Coverage (MS A–D)	AD vs HC	k = 4–6 per class (Table 1). Johansson 2025 [62] reports coverage only, and in mutation carriers rather than a clinically defined AD group.	Yes, Das 2026 [30]; Piton 2026 [31] for selected classes.	As above.	As for duration at this stage. Johansson 2025 [62] is additionally not commensurable because its groups are defined by APP/PSEN1/PSEN2 mutation status rather than by clinical diagnosis.
All temporal parameters	SCD vs HC	k = 0–1 per class (Table 1): a single counted contrast, microstate C, from Lassi 2023 [32]. Smailovic 2019 [35] contributes the largest SCD sample (n = 210) but fitted healthy-control grand-mean maps to the patient recordings and compared the patient groups only, so it yields no SCD-versus-control parameter contrast.	No. Neither meta-analysis pooled the SCD stage.	Not applicable, a single contributing contrast.	Below the pre-specified feasibility threshold of three studies by a wide margin: one contributing contrast, for one microstate class. Pooling is arithmetically impossible, not merely inadvisable.
Transition probabilities	MCI vs HC and AD vs HC	Five publications report transition probabilities: Schumacher 2020 [49], Lin 2021 [55], Lian 2021 [47], Lassi 2023 [32], Hou 2025 [51] (OpenNeuro cluster).	No. Neither meta-analysis pooled transition probabilities.	Reported as matrices rather than as single effect estimates; dispersion statistics for individual transitions were not recorded during extraction.	A transition probability is defined between an ordered pair of classes, so pooling requires that the class labels of both members of the pair correspond across studies. Post hoc re-sorting of template maps is not possible from published matrices, so fewer than three studies yield a commensurable estimate for any single transition.
Sequence / complexity entropy	MCI vs HC and AD vs HC	Four publications, each using a different metric: Tait 2020 [48] (Lempel–Ziv complexity of the transition sequence); Wu 2025 [64] (PCA-reduced entropy measures); Wan 2024 [60] (microstate sequence non-randomness index); Nobukawa 2024 [59] (instantaneous phase-based state transitions).	No.	Not applicable, no two publications report the same quantity, so no common estimate exists to weight.	The four metrics are not different estimators of one quantity but four distinct constructs, so no two studies can enter a common pooled estimate. The feasibility threshold of three studies reporting an identical parameter is met by none of them.
All temporal parameters	MCI vs AD (direct contrast)	Direct within-continuum contrasts are reported by a subset of the multigroup studies: Musaeus 2019 [46], Musaeus 2020 [33], Lian 2021 [47], Smailovic 2019 [35].	Partial, a within-continuum estimate is available within Das 2026 [30].	Not recorded separately from the versus-control contrasts during extraction.	Fewer than three studies report the same class-by-parameter contrast on a common scale; Smailovic 2019 [35] reports its patient-group comparison as a gradient across stages rather than as a pairwise MCI-versus-AD effect. The within-continuum estimate in Das 2026 [30] was used as a contextual benchmark instead.

Abbreviations: MS, microstate; HC, healthy controls; MCI, mild cognitive impairment; AD, Alzheimer's disease; SCD, subjective cognitive decline; LZC, Lempel–Ziv complexity; SD, standard deviation; CI, confidence interval.

Note on double counting. Re-analysing the primary studies included in this review would not itself constitute double counting. Double counting arises only where the same data enter a single pooled estimate more than once, which is not the case when primary studies are pooled afresh (Section 2.7). For the outcomes already covered by Das et al. (2026) [30] and Piton et al. (2026) [31], the constraint on an independent pooling is therefore one of limited incremental value, not of validity. Double counting is a risk in this review only in the distinct sense addressed by the two-tier evidence structure (Section 2.7): treating the pooled results of the two meta-

analyses as evidence additional to the same primary studies from which those results were derived. An earlier version of this table described re-pooling itself as double counting; that wording was inconsistent with Section 2.7 and has been corrected.

Note on the feasibility threshold. The pre-specified threshold for an independent pooled analysis was three or more studies reporting an identical microstate parameter for an identical diagnostic group comparison with sufficient extractable numerical data (Section 2.7). The final column above states, for each outcome, which element of that threshold was not met: the number of contributing studies (SCD vs HC; sequence and complexity entropy; MCI vs AD), the identity of the parameter across studies (transition probabilities; entropy metrics), or the commensurability of the reported scale (ratio-normalised, alpha-band-restricted, PCA-transformed and derived-index values). For the six duration, occurrence and coverage outcomes at the MCI and AD dementia stages the threshold was met in study number, and the reason for not pooling is the incremental-value argument set out in Section 2.7 together with the class-label non-equivalence described above.

Note on the number of commensurable studies. Because several of the principal comparisons are supported by more than three primary studies, the decision not to pool cannot rest on study number alone. Supplementary Table S8 therefore reports, for every included study, the segmentation algorithm, the number of microstate classes extracted, and the form in which parameters are reported. Of the 26 studies, 18 extracted a four-class solution; one extracted five classes, two extracted seven, one reported both four- and seven-class solutions, and four did not report the number extracted. After excluding studies whose reported quantities are not the canonical temporal parameters, are restricted to a single frequency band, are ratio-normalised, are PCA-transformed, or are derived indices, nine studies remain that could enter a common pooled estimate in principle: four of these contribute an MCI-versus-control contrast and eight an AD-versus-control contrast, distributed across four microstate classes and three parameters. For the AD-versus-control comparison the three-study threshold is therefore met in several cells on commensurability grounds alone, and the reason for not pooling there is the one given in Section 2.7: the class labels of studies using different clustering families cannot be aligned post hoc from published summary statistics, and aggregate estimates over substantially the same cohorts are already published.

Table S6. Citation matrix quantifying overlap in primary studies between the two contextual meta-analyses and the present review.

Inclusion in each meta-analysis was determined from the studies each publication lists as contributing to its quantitative synthesis (Das et al. 2026, Section 3; Piton et al. 2026, Section 3.1 and Tables 1-2).

#	Primary study (as listed in Supplementary Table S1)	In Das et al. 2026 [30] (1 = yes, 0 = no)	In Piton et al. 2026 [31] (1 = yes, 0 = no)	In present review (1 = yes)	Times counted (row total)
B1	Strik et al., 1997 ‡ [53]	1	0	1	2
B2	Dierks et al., 1997 ‡ [52]	1	0	1	2
B3	Stevens & Kircher, 1998 [54]	0	0	1	1
B4	Nishida et al., 2013 [50]	1	1	1	3
B5	Schumacher et al., 2020 [49]	1	1	1	3
B6	Musaeus et al., 2019 [46]	1	1	1	3
B7	Smailovic et al., 2019 [35]	1	0	1	2
B8	Tait et al., 2020 [48]	1	0	1	2
B9	Musaeus et al., 2020 [33]	1	1	1	3
B10	Lassi et al., 2023 [32]	1	1	1	3
B11	Lin et al., 2021 [[55]	1	0	1	2
B12	Lian et al., 2021 [47]	1	1	1	3
B13	Teipel et al., 2021 [56]	1	1	1	3
B14	Yan et al., 2024 [34]	1	1	1	3
B15	Bejia et al., 2023 [57]	1	0	1	2
B16	Li Z et al., 2024 [58]	1	1	1	3
B17	Nobukawa et al., 2024 [59]	1	0	1	2
B18	Wan et al., 2024 † [60]	0	0	1	1
B19	Yang et al., 2024 † [61]	0	0	1	1
B20	Wu et al., 2025 [64]	1	0	1	2
B21	Tarailis et al., 2025 [63]	1	1	1	3
B22	Johansson et al., 2025 [62]	0	0	1	1
B23	Chang, 2025 † [65]	1	0	1	2
B24	Hou et al., 2025 † [51]	1	0	1	2
B25	Lv et al., 2026 [66]	0	0	1	1
B26	Sayman et al., 2026 [67]	0	0	1	1
	Number of primary studies (r)				26
	Number of evidence sources compared (c)				3
	Total inclusions across sources (N)				56
	Corrected Covered Area, CCA = (N - r) / (r x c - r)				0.577 (57.7%)
	Overlap interpretation (Pieper et al. 2014): CCA 0-5 slight; 6-10 moderate; 11-15 high; >15 very high				

Method. The primary studies contributing to each meta-analysis were extracted from the reference list of the respective publication and cross-tabulated against the primary studies included in the present review. Overlap was quantified using the corrected covered area (CCA), the standard measure of primary-study overlap between evidence syntheses. Because both meta-analyses draw on a shared pool of primary studies, concordance between their pooled estimates is reported in the manuscript as consistency arising from shared source data, not as independent replication (Sections 2.7, 3.3.2, 3.3.3, 4.2).

Table S7. Sensitivity analysis: eligibility of each included study under the original a priori list of diagnostic frameworks (NIA-AA 2011/2018, DSM-5, ICD-10/11 only), before the amendment described in Section 2.1 of the manuscript.

#	Study	Diagnostic framework recorded (Table S1)	Eligible under the ORIGINAL	Basis for the judgement

			criteria?	
B1	Strik et al., 1997 [53]	NINCDS-ADRDA	**No**	NINCDS-ADRDA only
B2	Dierks et al., 1997 [52]	NINCDS-ADRDA	**No**	NINCDS-ADRDA only
B3	Stevens & Kircher, 1998 [54]	SIDAM	**No**	SIDAM only
B4	Nishida et al., 2013 [50]	DSM-IV	**No**	DSM-IV only
B5	Schumacher et al., 2020 [49]	McKhann 2011 (= NIA-AA 2011) + McKeith 2005	**Yes**	McKhann et al. 2011 is the NIA-AA 2011 dementia criterion (ref [10])
B6	Musaeus et al., 2019 [46]	NIA-AA 2011	**Yes**	NIA-AA 2011
B7	Smailovic et al., 2019 [35]	NIA-AA 2011	**Yes**	NIA-AA 2011
B8	Tait et al., 2020 [48]	DSM-IV + NINCDS-ADRDA; IWG-2	**No**	no framework on the original list
B9	Musaeus et al., 2020 [46]	NIA-AA 2011	**Yes**	NIA-AA 2011
B10	Lassi et al., 2023 [32]	NIA-AA 2018 (MCI); SCD-I (SCD group)	**Yes**	NIA-AA 2018 but SCD arm rests on SCD-I
B11	Lin et al., 2021 [55]	IWG-II; Neary/McKhann 2001	**No**	IWG-II only
B12	Lian et al., 2021 [47]	NIA-AA 2011	**Yes**	NIA-AA 2011
B13	Teipel et al., 2021 [56]	NINCDS-ADRDA + NIA-AA 2011	**Yes**	NIA-AA 2011 also applied
B14	Yan et al., 2024 [61]	NIA-AA 2011	**Yes**	NIA-AA 2011
B15	Bejia et al., 2023 [57]	not reported	**No**	no named validated framework
B16	Li Z et al., 2024 [58]	NIA-AA (AD); MCI not reported	**Yes**	NIA-AA named for the AD group
B17	Nobukawa et al., 2024 [59]	NINCDS-ADRDA + DSM-IV	**No**	no framework on the original list
B18	Wan et al., 2024 [60]	DSM-IIIR/IV + NINCDS-ADRDA	**No**	no framework on the original list
B19	Yang et al., 2024 [61]	NIA-AA (dataset labels)	**Yes**	NIA-AA
B20	Wu et al., 2025 [64]	Petersen / NIA-AA	**Yes**	NIA-AA
B21	Tarailis et al., 2025 [63]	MoCA screening; formal criteria not reported	**No**	no named validated framework
B22	Johansson et al., 2025 [62]	monogenic mutation status (APP/PSEN1/PSEN2)	**No**	not a listed clinical framework
B23	Chang, 2025 [65]	NINCDS-ADRDA; DSM-IIIR/IV; ICD-10	**Yes**	ICD-10 is on the original list
B24	Hou et al., 2025 [51]	NINCDS-ADRDA (per source dataset)	**No**	NINCDS-ADRDA only
B25	Lv et al., 2026 [66]	NINCDS-ADRDA	**No**	NINCDS-ADRDA only
B26	Sayman et al., 2026 [67]	Petersen; DSM-5 Mild NCD	**Yes**	DSM-5 is on the original list
	**Total (n = 26)**		**Yes = 13; No = 13**	

Note. The original a priori criteria (Section 2.2) accepted NIA-AA 2011 or 2018, DSM-5, and ICD-10 or ICD-11. The amendment reported in Section 2.1 added NINCDS-ADRDA, DSM-IV, IWG-II, SIDAM and the SCD-I research criteria. Schumacher et al. (2020) is classified as eligible under the original criteria because McKhann et al. (2011), the criterion it applies, is the NIA-AA 2011 dementia criterion (manuscript reference [10]). Chang (2025) and Sayman et al. (2026) are eligible because they additionally applied ICD-10 and DSM-5 respectively. Lassi et al. (2023) is eligible on the basis of NIA-AA 2018 for its MCI group, but its SCD group was defined using the SCD-I criteria and would not have been eligible under the original list; the consequences are reported in Section 3.1. The second amendment (comparator relaxed from age- and sex-matched to age-matched controls) altered no inclusion decision and is therefore not tabulated here.

Table S8. Analytical parameters bearing on cross-study commensurability, per included study. Algorithm, number of classes and reported parameters are as recorded in Supplementary Table S1.

#	Study	Segmentation algorithm	Classes extracted	Form in which parameters are reported	Consequence for inclusion in a common pooled estimate
B1	Strik et al., 1997 [53] ‡	Modified K-means (Lehmann)	4	Duration, occurrence, coverage-absolute	Companion report of the same cohort as B2; contributes one independent sample.
B2	Dierks et al., 1997 [52] ‡	Modified K-means (Lehmann)	4	Duration, occurrence, coverage-absolute	Companion report of the same cohort as B1.
B3	Stevens & Kircher, 1998 [54]	Adaptive segmentation (Lehmann)	Not reported	Duration, number of microstates, EEG reactivity	Number of classes not reported and the reported quantities are not the

					canonical temporal parameters; no class-level contrast can be aligned.
B4	Nishida et al., 2013 [50]	Modified K-means	4	Duration, occurrence, coverage-absolute	Poolable in principle for a 4-class solution, subject to class-label correspondence.
B5	Schumacher et al., 2020 [49]	TAAHC	5	Duration, occurrence, coverage, transition probability	Five-class solution: classes do not map one-to-one onto a four-class solution, so parameters cannot enter a common pooled estimate.
B6	Musaeus et al., 2019 [46]	Modified K-means	4	Duration, occurrence, coverage-absolute	Poolable in principle, subject to class-label correspondence.
B7	Smailovic et al., 2019 [35]	Modified K-means	4	Duration, occurrence, coverage-absolute	Healthy-control grand-mean maps fitted to patient recordings; patient groups compared with one another only, so no versus-control contrast is available.
B8	Tait et al., 2020 [48]	Modified K-means (k-means++ initialisation)	4	Duration, coverage, Lempel–Ziv complexity of the transition sequence	Complexity metric is a distinct construct reported by no other study; temporal parameters poolable in principle.
B9	Musaeus et al., 2020 [33]	Modified K-means	4	Duration, occurrence, coverage-absolute	Poolable in principle, subject to class-label correspondence.
B10	Lassi et al., 2023 [32]	AAHC	4	Duration, occurrence, coverage, transition probability	Different clustering family from modified K-means; template maps are not guaranteed to correspond, so labels require re-sorting that cannot be performed post hoc.
B11	Lin et al., 2021 [55]	K-means (EEGLAB Microstate plug-in)	4	Duration, occurrence, GFP peaks per second, transition probability	Occurrence expressed as GFP peaks per second rather than events per second; not the same unit as the other studies.
B12	Lian et al., 2021 [47]	AAHC	4	Duration, occurrence, coverage, transition probability	As for B10: different clustering family.
B13	Teipel et al., 2021 [56]	Modified K-means (EEGLAB Microstate plug-in)	4 (selected)	Duration, occurrence, coverage; microstate topologies related to fMRI networks	Poolable in principle; primary reporting is oriented to the fMRI comparison.
B14	Yan et al., 2024 [34]	Modified K-means	4	Duration, occurrence, coverage-absolute	Poolable in principle, subject to class-label correspondence.
B15	Bejia et al., 2023 [57]	Not reported	Not reported	Duration, occurrence, coverage plus spectral and connectivity measures	Neither algorithm nor number of classes reported; commensurability cannot be established.
B16	Li Z et al., 2024 [58]	Not reported	Not reported	Duration, occurrence, brain homeostasis coefficient	Principal outcome is a derived index on its own scale; algorithm and class number not reported.
B17	Nobukawa et al., 2024 [59]	Instantaneous-phase microstate analysis	4	Instantaneous phase-based state transitions	States are defined by leading phase location rather than by scalp topography; not the same construct.
B18	Wan et al., 2024 [60] †	CEEMD-enhanced modified K-means	4	Microstate sequence non-randomness index	Single derived index; no canonical temporal parameter reported. OpenNeuro cluster.
B19	Yang et al., 2024 [61] †	Not reported	Not reported	Alpha-band microstate features (duration, occurrence, coverage)	Parameters restricted to the alpha band, not broadband; not on the same scale as the other studies. OpenNeuro cluster.
B20	Wu et al., 2025 [64]	Modified K-means	4	Duration, coverage, entropy measures; PCA applied	Parameters entered analysis as principal components rather than as raw values.
B21	Tarailis et al., 2025 [63]	High-density EEG microstate analysis	4 (A–D)	Duration, occurrence, coverage; mind-wandering correlates	Poolable in principle; comparator is a healthy older group within a three-group design.
B22	Johansson et al., 2025 [62]	Not reported	7	Coverage only	Seven-class solution and coverage alone; groups defined by APP/PSEN1/PSEN2 mutation status rather than clinical diagnosis.
B23	Chang, 2025 [65] †	Modified K-means (MICROSTATELAB)	4 and 7	Duration, occurrence, coverage-ratio-normalised	Values are ratio-normalised rather than absolute, and two class solutions are reported. OpenNeuro cluster.

B24	Hou et al., 2025 [51] †	Modified K-means	4	Coverage, duration, occurrence, transition probabilities	Poolable in principle. OpenNeuro cluster.
B25	Lv et al., 2026 [66]	Modified K-means	4	Duration, occurrence, coverage-absolute	Poolable in principle, subject to class-label correspondence. BrainLat cluster of one.
B26	Sayman et al., 2026 [67]	K-means (MICROSTATELAB; Metamaps2023 templates)	7	Global explained variance, duration, occurrence, coverage	Seven-class solution fitted to published template maps; classes do not map one-to-one onto a four-class solution.

Note. "Poolable in principle" means only that the study reports a canonical temporal parameter on an absolute scale from a four-class solution; it does not imply that the summary statistics required for inverse-variance weighting were reported, which was not recorded during extraction (Table S5). Studies marked † belong to the shared OpenNeuro cluster and ‡ to the Strik/Dierks companion cohort; within each cluster the constituent publications contribute a single independent sample (Section 2.7).

Table S9. Cell-by-cell attribution of the direction assignments in Table 1, with the verification status of each assignment.

Table 1 cell	Independent sample	Study	Direction	Verified against the primary report?	Basis for the assignment
A/SCD	—	—	k = 0	—	No independent sample contributes a contrast for this cell.
A/MCI	B12	Lian et al., 2021 [47]	no difference	Yes	Table 2: duration p=0.066, occurrence p=0.64, coverage p=0.11
A/MCI	B20	Wu et al., 2025 [64]	↓ decrease	No — as recorded in Table S1	Supplementary Table S1 (not re-verified)
A/MCI	B21	Tarailis et al., 2025 [63]	↑ increase	Yes	Results: MCI vs healthy older, GEV p=0.011, dur p=0.033, cov p=0.015, occ p=0.031
A/MCI	B6	Musaeus et al., 2019 [46]	↑ increase	Yes	Table 4 + post hoc: occurrence p=0.0411, coverage p=0.0077 vs HC
A/MCI	B9	Musaeus et al., 2020 [33]	↑ increase	No — as recorded in Table S1	Supplementary Table S1 (not re-verified)
A/AD	B12	Lian et al., 2021 [47]	no difference	Yes	Table 2: as above
A/AD	B14	Yan et al., 2024 [34]	no difference	Yes	Yan et al. 2024 — Table 2: duration p_FDR=0.123, occurrence p_FDR=0.185, coverage p_FDR=0.765
A/AD	OpenNeuro cluster	Chang, 2025 [65]	not counted	Yes	Chang (2025) reports no significant difference in the four-class solution, but Hou et al. (2025) reports that 21 of 24 microstate features differed; the cluster is recorded as inconsistent and contributes no vote for this cell (Section 2.7).
A/AD	BrainLat cluster	Lv et al., 2026 [66]	no difference	Yes	Lv et al. 2026 — Table 3: no significant HC-vs-AD post hoc contrast for any microstate A parameter
A/AD	B4	Nishida et al., 2013 [50]	no difference	Yes	Table 2: duration p=0.350, occurrence p=0.643
A/AD	B5	Schumacher et al., 2020 [49]	no difference	No — as recorded in Table S1	Supplementary Table S1 (not re-verified)
A/AD	B6	Musaeus et al., 2019 [46]	↑ increase	Yes	Table 4 + post hoc: occurrence p=0.0395, coverage p=0.0066 vs HC
A/AD	B9	Musaeus et al., 2020 [33]	↑ increase	No — as recorded in Table S1	Supplementary Table S1 (not re-verified)
B/SCD	—	—	k = 0	—	No independent sample contributes a contrast for this cell.
B/MCI	B12	Lian et al., 2021 [47]	no difference	Yes	Table 2 post hoc: duration HC-MCI p=1.00, coverage HC-MCI p=1.00
B/MCI	B16	Li Z et al., 2024 [58]	↑ increase	No — as recorded in Table S1	Supplementary Table S1 (not re-verified)
B/MCI	B20	Wu et al., 2025 [64]	↓ decrease	No — as recorded in Table S1	Supplementary Table S1 (not re-verified)
B/MCI	B21	Tarailis et al., 2025 [63]	no difference	Yes	No significant contrast reported for microstate B
B/MCI	B26	Sayman et al., 2026 [67]	↑ increase	No — as recorded in Table S1	Supplementary Table S1 (not re-verified)
B/MCI	B6	Musaeus et al., 2019 [46]	no difference	Yes	Table 4: duration p=0.082, occurrence p=0.422, coverage p=0.26
B/AD	B11	Lin et al., 2021 [55]	↑ increase	No — as recorded	Supplementary Table S1 (not re-verified)

				in Table S1	
B/AD	B12	Lian et al., 2021 [47]	↑ increase	Yes	Table 2 post hoc: duration HC-AD p=0.008, coverage HC-AD p=0.007
B/AD	B14	Yan et al., 2024 [34]	no difference	Yes	Yan et al. 2024 — Table 2: occurrence p=0.015 but p_FDR=0.075; duration p_FDR=0.166; coverage p_FDR=0.782
B/AD	OpenNeuro cluster	Chang, 2025 [65]	↑ increase	Yes	Table 2 + Fig 2: CTL significantly lower duration/coverage than AD, p<0.001
B/AD	BrainLat cluster	Lv et al., 2026 [66]	no difference	Yes	Lv et al. 2026 — Table 3: no significant HC-vs-AD post hoc contrast for any microstate B parameter
B/AD	B4	Nishida et al., 2013 [50]	no difference	Yes	Table 2: duration p=0.247, occurrence p=0.428
B/AD	B5	Schumacher et al., 2020 [49]	no difference	No — as recorded in Table S1	Supplementary Table S1 (not re-verified)
B/AD	B6	Musaeus et al., 2019 [46]	no difference	Yes	Table 4: as above
C/SCD	B10	Lassi et al., 2023 [32]	↓ decrease	No — as recorded in Table S1	Supplementary Table S1 (not re-verified)
C/MCI	B10	Lassi et al., 2023 [32]	↓ decrease	No — as recorded in Table S1	Supplementary Table S1 (not re-verified)
C/MCI	B12	Lian et al., 2021 [47]	no difference	Yes	Table 2 post hoc: coverage HC-MCI p=0.92
C/MCI	B20	Wu et al., 2025 [64]	↑ increase	No — as recorded in Table S1	Supplementary Table S1 (not re-verified)
C/MCI	B21	Tarailis et al., 2025 [63]	no difference	Yes	Significant microstate C contrasts are vs younger controls only; MCI vs age-matched older not significant
C/MCI	B26	Sayman et al., 2026 [67]	↑ increase	No — as recorded in Table S1	Supplementary Table S1 (not re-verified)
C/MCI	B6	Musaeus et al., 2019 [46]	no difference	Yes	Table 4: duration p=0.725, occurrence p=0.256, coverage p=0.578
C/AD	B12	Lian et al., 2021 [47]	no difference	Yes	Table 2 post hoc: coverage HC-AD p=0.25
C/AD	B14	Yan et al., 2024 [34]	↑ increase	Yes	Yan et al. 2024 — Table 2: duration AD 0.069 vs HC 0.063, p=0.016, p_FDR=0.040 (the only parameter surviving FDR)
C/AD	OpenNeuro cluster	Chang, 2025 [65]	not counted	Yes	Chang (2025) reports no significant difference in the four-class solution, but Hou et al. (2025) reports that 21 of 24 microstate features differed; the cluster is recorded as inconsistent and contributes no vote for this cell (Section 2.7).
C/AD	BrainLat cluster	Lv et al., 2026 [66]	↑ increase	Yes	Lv et al. 2026 — Table 3: duration p=0.002, occurrence p<0.001, coverage p<0.001; Table 2 means all higher in AD
C/AD	B4	Nishida et al., 2013 [50]	no difference	Yes	Table 2: duration p=0.398, occurrence p=0.818
C/AD	B5	Schumacher et al., 2020 [49]	no difference	No — as recorded in Table S1	Supplementary Table S1 (not re-verified)
C/AD	B6	Musaeus et al., 2019 [46]	no difference	Yes	Table 4: as above
D/SCD	—	—	k = 0	—	No independent sample contributes a contrast for this cell.
D/MCI	B12	Lian et al., 2021 [47]	no difference	Yes	Table 2: duration p=0.36, occurrence p=0.19, coverage p=0.11
D/MCI	B20	Wu et al., 2025 [64]	↓ decrease	No — as recorded in Table S1	Supplementary Table S1 (not re-verified)
D/MCI	B21	Tarailis et al., 2025 [63]	no difference	Yes	No significant contrast reported for microstate D
D/MCI	B6	Musaeus et al., 2019 [46]	no difference	Yes	Table 4: duration p=0.55, occurrence p=0.304, coverage p=0.307
D/AD	B12	Lian et al., 2021 [47]	no difference	Yes	Table 2: as above
D/AD	B14	Yan et al., 2024 [34]	no difference	Yes	Yan et al. 2024 — Table 2: duration p=0.014 but p_FDR=0.070; occurrence p_FDR=0.483; coverage p_FDR=0.532
D/AD	OpenNeuro cluster	Chang, 2025 [65]	↓ decrease	Yes	Table 2 + Fig 2: occurrence significantly higher in CTL than AD, p<0.001
D/AD	BrainLat cluster	Lv et al., 2026 [66]	↓ decrease	Yes	Lv et al. 2026 — Table 3: occurrence p=0.028, coverage p=0.018; Table 2 means lower in AD
D/AD	B4	Nishida et al., 2013 [50]	no difference	Yes	Table 2: duration p=0.336, occurrence p=0.267
D/AD	B5	Schumacher et al., 2020 [49]	no difference	No — as recorded in Table S1	Supplementary Table S1 (not re-verified)
D/AD	B6	Musaeus et al., 2019	no	Yes	Table 4: as above

		[46]	difference		
withdrawn	—	Teipel et al. 2021 [56]	—	Yes	Microstates are labelled 1-4 and are not assigned canonical A-D labels; the significant contrasts are occurrence of microstate 2 (increased) and microstate 3 (decreased), p=0.02 each, with no significant duration difference. The reported increase in frontal network expression is a resting-state fMRI spatial component, not a microstate parameter.
withdrawn	—	Johansson et al. 2025 [62]	—	Yes	A seven-class solution is used throughout and the report states explicitly that its class B correlates only poorly to moderately with four-class class B maps. Groups are defined by APP/PSEN1/PSEN2 mutation status rather than by clinical diagnosis, and the symptomatic group comprises seven participants.

Note. Seven primary reports were re-examined in full against the direction assignments recorded in Supplementary Table S1: Nishida et al. (2013) [50], Musaeus et al. (2019) [46], Lian et al. (2021) [47], Teipel et al. (2021) [56], Tarailis et al. (2025) [63], Johansson et al. (2025) [62] and Chang (2025) [65]. Eight assignments were corrected and two studies were withdrawn from the class-level counts. Assignments marked "No" rest on the outcome summaries in Supplementary Table S1 and have not been re-derived from the primary report; they are reported here so that the provenance of every count in Table 1 is explicit. Because null results are seldom stated in a summary of key findings, the no-difference counts in Table 1 rose from 12 to 21 on re-examination, and remain a lower bound.
