# Supplementary material for: Resting-State EEG Microstate Dynamics as Neurophysiological Biomarkers Across the Alzheimer’s Disease Continuum: A Systematic Review
Source: Diagnostics (Basel). 2026 Sep 17;16(18):3019. doi: 10.3390/diagnostics16183019 (PMC13605323; doi:10.3390/diagnostics16183019)
Supplement: Supplementary file 1 [file diagnostics-16-03019-s001.zip › PRISMA.2020.Checklist.v5.pdf]

PRISMA 2020 Checklist

Resting-State EEG Microstate Dynamics as Neurophysiological Biomarkers Across the Alzheimer's Disease Continuum: A Systematic Review

Manuscript ID: diagnostics-4539682 · Simfukwe C., An S.S.A., Youn Y.C.

Section and Topic	Item #	Checklist item	Location where item is reported
**TITLE**			
**Title**	1	Identify the report as a systematic review.	Title (page 1): "… A Systematic Review". Also flagged as "Systematic Review Paper" in the running head.
**ABSTRACT**			
**Abstract**	2	See the PRISMA 2020 for Abstracts checklist.	Abstract (page 1), structured under Background/Objectives, Methods, Results, Conclusions.
**INTRODUCTION**			
**Rationale**	3	Describe the rationale for the review in the context of existing knowledge.	Section 1 (Introduction); Sections 1.1–1.2, and Section 1.3 (Rationale and Aim of the Review).
**Objectives**	4	Provide an explicit statement of the objective(s) or question(s) the review addresses.	Section 1.3 (Rationale and Aim of the Review); restated in Abstract, Background/Objectives.
**METHODS**			
**Eligibility criteria**	5	Specify the inclusion and exclusion criteria for the review and how studies were grouped for the syntheses.	Section 2.2 (Eligibility Criteria), specified using the PICO framework; grouping of studies for the syntheses is described in Section 2.7. Two amendments were made to the eligibility criteria after the initial searches and are reported in Section 2.1; their effect on study selection is assessed in a sensitivity analysis

Section and Topic	Item #	Checklist item	Location where item is reported
			reported in Section 3.1 and Supplementary Table S7.
**Information sources**	6	Specify all databases, registers, websites, organisations, reference lists and other sources searched or consulted to identify studies. Specify the date when each source was last searched or consulted.	Section 2.3 (Information Sources and Search Strategy) and Supplementary Table S3. Bibliographic databases: PubMed/MEDLINE and EMBASE, covering 1 January 1990 to 30 June 2026 and last searched on 30 June 2026; Scopus, IEEE Xplore and Web of Science Core Collection, each last searched on 5 August 2026. Registers: PROSPERO, ClinicalTrials.gov and the WHO International Clinical Trials Registry Platform, each last searched on 5 August 2026. Other sources: the reference lists of all included studies and of relevant systematic reviews identified during screening were hand-searched. PsycINFO was not searched, as it is not held under the authors' institutional subscription; the prepared strategy is retained in Supplementary Table S3, Section F, and the omission is acknowledged in Section 4.5.
**Search strategy**	7	Present the full search strategies for all databases, registers and websites, including any filters and limits used.	Section 2.3 and Supplementary Table S3, which reports every strategy verbatim: PubMed/MEDLINE (Section A) and EMBASE (Section B), with the full Boolean structure, field tags, MeSH and Emtree controlled vocabulary, the human-species filter and the

Section and Topic	Item #	Checklist item	Location where item is reported
			1990–2026 date limit, final search 30 June 2026; Web of Science Core Collection (Section D), Scopus (Section E), IEEE Xplore (Section G) and the three registration sources (Section H), each searched on 5 August 2026, with source-specific strategies, execution dates and record yields; and Section I, the revised overall yield after the database expansion. Web of Science Core Collection was searched and retrieved 145 records, of which 30 duplicated records already identified; four records were assessed at full text and all four were excluded (Section 3.1). PsycINFO was not searched; its prepared strategy is retained in Supplementary Table S3, Section F, for use in future updates of the review (Section 4.5).
**Selection process**	8	Specify the methods used to decide whether a study met the inclusion criteria of the review, including how many reviewers screened each record and each report retrieved, whether they worked independently, and if applicable, details of automation tools used in the process.	Section 2.4 (Study Selection): two reviewers, independent blinded two-stage screening in Rayyan; third-reviewer adjudication; Cohen's κ reported.
**Data collection process**	9	Specify the methods used to collect data from reports, including how many reviewers collected data from each report, whether they worked independently, any processes for obtaining or confirming data from study investigators, and if applicable, details of automation tools used in the process.	Section 2.5 (Data Extraction): piloted standardised form, two reviewers extracting independently, discrepancies resolved by consensus and cross-verified against source documents.
**Data items**	10a	List and define all outcomes for which data were sought. Specify whether all results that were compatible with each outcome domain in each study were sought, and if not, the methods used to decide which results to collect.	Section 2.5: microstate outcomes defined (duration, occurrence, coverage, transition

Section and Topic	Item #	Checklist item	Location where item is reported
			probability, syntax entropy) per class; Section 2.2 specifies required outcomes.
	10b	List and define all other variables for which data were sought. Describe any assumptions made about any missing or unclear information.	Section 2.5: study characteristics, participant demographics, diagnostic criteria, EEG acquisition parameters, microstate analysis parameters, statistical findings, and ATN biomarker correlates.
**Study risk of bias assessment**	11	Specify the methods used to assess risk of bias in the included studies, including details of the tool(s) used, how many reviewers assessed each study and whether they worked independently, and if applicable, details of automation tools used in the process.	Section 2.6 (Risk of Bias and Methodological Quality Assessment): NOS-CS applied independently by two reviewers; scoring bands defined; domain-level results in Supplementary Table S2. The two contextual meta-analyses were appraised separately using AMSTAR 2 (Supplementary Table S4).
**Effect measures**	12	Specify for each outcome the effect measure(s) used in the synthesis or presentation of results.	Section 2.7: standardised mean difference (SMD / Hedges' g) as the effect measure for pooled estimates; effect directions tabulated (Table 1).
**Synthesis methods**	13a	Describe the processes used to decide which studies were eligible for each synthesis.	Section 2.7: the synthesis is organised by clinical stage, microstate class and parameter type, with a feasibility threshold of three or more studies specified for any pooling. A two-tier evidence structure separates the 26 included primary studies from the two contextual meta-analyses; the overlap in primary studies between the two meta-analyses and the present review is quantified in

Section and Topic	Item #	Checklist item	Location where item is reported
			Supplementary Table S6 (corrected covered area 57.7%). A pre-specified shared-cohort clustering rule determines the number of independent samples contributing to each outcome (Section 2.7; cluster membership in Supplementary Table S1).
	13b	Describe any methods required to prepare the data for presentation or synthesis, such as handling of missing summary statistics, or data conversions.	Section 2.7: narrative synthesis following SWiM guidance; conditional pooling criteria and data requirements described.
	13c	Describe any methods used to tabulate or visually display results of individual studies and syntheses.	Section 2.7 and Section 3.3: effect-direction consistency tabulated (Table 1); study characteristics in Supplementary Table S1.
	13d	Describe any methods used to synthesize results and provide a rationale for the choice(s). If meta-analysis was performed, describe the model(s), method(s) to identify statistical heterogeneity, and software package(s) used.	Section 2.7: a random-effects model with I^2 and Cochran's Q was pre-specified as conditional on a feasibility threshold of three or more studies reporting an identical parameter for an identical comparison with sufficient extractable data. That threshold was not met in a form permitting a valid independent pooling, so a structured narrative synthesis following SWiM guidance was used. A per-outcome account of pooling feasibility is given in Supplementary Table S5, and the analytical parameters bearing on cross-study commensurability are tabulated per study in Supplementary Table S8. Pooled estimates from the two contextual meta-analyses are reported as external benchmarks

Section and Topic	Item #	Checklist item	Location where item is reported
			only and were not combined with the primary-study data.
	13e	Describe any methods used to explore possible causes of heterogeneity among study results.	Section 2.7 and Section 3.3.4 (Heterogeneity and Inconsistent Findings): heterogeneity examined narratively across electrode density, algorithm, and diagnostic criteria.
	13f	Describe any sensitivity analyses conducted to assess robustness of the synthesized results.	Section 2.1 and Section 3.1: no de novo quantitative meta-analysis was performed, so no statistical sensitivity analysis of a pooled estimate was possible. A sensitivity analysis of study selection was conducted, in which the original a priori list of diagnostic frameworks was reapplied to all 26 included studies to assess the effect of the eligibility amendments (Section 2.1); the method is stated in Section 2.1 and the study-by-study classification in Supplementary Table S7.
**Reporting bias assessment**	14	Describe any methods used to assess risk of bias due to missing results in a synthesis (arising from reporting biases).	Section 2.6: no quantitative method for assessing risk of bias due to missing results (funnel-plot asymmetry, Egger's test) was pre-specified, as such methods require a pooled analysis. Sections 2.3 and 3.1: reference-list hand-searching, no language restriction, and searches of three registries were used to reduce retrieval bias; no eligible unpublished or ongoing studies were identified.

Section and Topic	Item #	Checklist item	Location where item is reported
**Certainty assessment**	15	Describe any methods used to assess certainty (or confidence) in the body of evidence for an outcome.	Section 2.6: no formal certainty-of-evidence framework (e.g., GRADE) was applied. NOS-CS quality scores and cross-study consistency were used only to inform narrative statements about confidence in the evidence (Sections 4.1, 4.5). AMSTAR 2 confidence ratings for the two contextual meta-analyses are reported in Supplementary Table S4.
**RESULTS**			
**Study selection**	16a	Describe the results of the search and selection process, from the number of records identified in the search to the number of studies included in the review, ideally using a flow diagram.	Section 3.1 (Study Selection) with full record counts; PRISMA 2020 flow diagram (Figure 2).
	16b	Cite studies that might appear to meet the inclusion criteria, but which were excluded, and explain why they were excluded.	Section 3.1: full-text exclusions are reported by category with counts, 45 in total across the original and expanded searches 35 at the original full-text stage, 6 from the Scopus, IEEE Xplore and registry searches, and 4 from the Web of Science Core Collection search. In addition, three records that had passed full-text assessment were excluded at re-screening against the a priori PICO criteria: one lacked a healthy-control comparator and two did not report an extractable microstate parameter. These are reported by reason and count in Section 3.1 and in the footnote to Supplementary Table S1. Individual citations

Section and Topic	Item #	Checklist item	Location where item is reported
			are not given for these or for the remaining category-level exclusions.
**Study characteristics**	17	Cite each included study and present its characteristics.	Section 3.2 (Study Characteristics and Quality Assessment); full details in Supplementary Table S1.
**Risk of bias in studies**	18	Present assessments of risk of bias for each included study.	Section 3.2.3 (Quality Assessment) summary; per-study NOS-CS scores in Supplementary Table S2.
**Results of individual studies**	19	For all outcomes, present, for each study: (a) summary statistics for each group and (b) an effect estimate and its precision, ideally using structured tables or plots.	Supplementary Table S1: per-study group sizes, diagnostic criteria, parameters reported, cognitive severity and direction of key findings versus controls. Group-level summary statistics with dispersion and study-level effect estimates with precision are NOT presented for every study: most primary studies did not report them, and their availability was judged during extraction at the level of the evidence base as a whole rather than recorded study by study (Sections 2.5, 4.5; Supplementary Table S5). The effect sizes with 95% CIs in Table 1 are pooled benchmarks from the two contextual meta-analyses, not per-study estimates. The analytical parameters bearing on whether studies are commensurable are reported per study in Supplementary Table S8. Every direction assignment in Table 1 was checked against the primary report in which it

Section and Topic	Item #	Checklist item	Location where item is reported
			originated; the cell-by-cell attribution and verification status are given in Supplementary Table S9.
**Results of syntheses**	20a	For each synthesis, briefly summarise the characteristics and risk of bias among contributing studies.	Section 3.3 (opening) and Sections 3.3.1–3.3.3; quality context in Section 3.2.3.
	20b	Present results of all statistical syntheses conducted. If meta-analysis was done, present for each the summary estimate and its precision and measures of statistical heterogeneity.	Sections 3.3.2–3.3.3: pooled Hedges' g estimates with 95% CIs and p-values from the two contextual meta-analyses are reported as external benchmarks; effect directions and effect sizes in Table 1. No de novo meta-analysis was performed.
	20c	Present results of all investigations of possible causes of heterogeneity among study results.	Section 3.3.4 (Heterogeneity and Inconsistent Findings).
	20d	Present results of all sensitivity analyses conducted to assess the robustness of the synthesized results.	Section 3.1: the sensitivity analysis reapplying the original a priori diagnostic-framework criteria is reported in full. Thirteen of the 26 included studies would have been ineligible under the original list and 13 retained; the direction of every principal finding at the MCI and AD dementia stages is unchanged, whereas the single SCD-versus-control contrast counted in Table 1 would not have been eligible. Study-by-study classification in Supplementary Table S7. No sensitivity analysis of a pooled estimate was possible, as no de novo meta-analysis was performed (Section 2.7).

Section and Topic	Item #	Checklist item	Location where item is reported
**Reporting biases**	21	Present assessments of risk of bias due to missing results (arising from reporting biases) for each synthesis assessed.	Section 4.5: no quantitative assessment of risk of bias due to missing results was performed. The potential under-representation of null findings is discussed narratively, informed by the registry search, which identified no eligible unpublished or ongoing studies.
**Certainty of evidence**	22	Present assessments of certainty (or confidence) in the body of evidence for each outcome assessed.	Sections 4.1 and 4.5: no formal outcome-level certainty ratings were generated. Confidence in the evidence is discussed narratively, informed by NOS-CS quality (Section 3.2.3) and the consistency descriptors in Table 1, which are explicitly stated not to constitute formal certainty ratings.
**DISCUSSION**			
**Discussion**	23a	Provide a general interpretation of the results in the context of other evidence.	Section 4.1 (Overview of Principal Findings) and Sections 4.2–4.3.
	23b	Discuss any limitations of the evidence included in the review.	Section 4.4 (Methodological Heterogeneity) and Section 4.5 (Limitations of Included Studies and of This Review).
	23c	Discuss any limitations of the review processes used.	Section 4.5 (Limitations of Included Studies and of This Review): the review was not prospectively registered and the internal protocol is not publicly accessible; a structured narrative synthesis was used in place of a de novo meta-analysis; the search covered five bibliographic databases and three registers but not PsycINFO; only English-language full texts

Section and Topic	Item #	Checklist item	Location where item is reported
			were assessed at the eligibility stage; eligibility criteria were amended after the initial searches (Section 2.1), with the effect assessed in Section 3.1; and the availability of summary statistics required for pooling was not recorded study by study.
	23d	Discuss implications of the results for practice, policy, and future research.	Section 4.6 (Clinical Translation and Future Directions) and Section 5 (Conclusion).
**OTHER INFORMATION**			
**Registration and protocol**	24a	Provide registration information for the review, including register name and registration number, or state that the review was not registered.	Section 2.1 (Protocol Registration and Reporting Standards): the review was not prospectively registered in a public registry.
	24b	Indicate where the review protocol can be accessed, or state that a protocol was not prepared.	Section 2.1: an a priori protocol covering eligibility criteria, search strategy and synthesis approach was prepared for internal use only. It was neither registered in a public registry nor published, and is therefore not publicly accessible.
	24c	Describe and explain any amendments to information provided at registration or in the protocol.	Section 2.1: two amendments were made to the a priori eligibility criteria after the initial searches were completed, and both are reported in Section 2.1. First, the list of acceptable diagnostic frameworks was broadened beyond NIA-AA, DSM-5 and ICD to include other internationally validated criteria applied in the source studies (NINCDS-ADRDA, DSM-IV, IWG-II, SIDAM and the SCD-I research

Section and Topic	Item #	Checklist item	Location where item is reported
			criteria), because the original list would otherwise have excluded the entire pre-2011 literature. Second, the comparator requirement was relaxed from an age- and sex-matched control group to an age-matched control group as the minimum. No changes were made to the index-test definition, the outcome definitions or the planned synthesis framework. Both amendments were applied uniformly to all records at re-screening. In addition, the information-source strategy was amended after the initial searches to add Scopus, IEEE Xplore, Web of Science Core Collection, PROSPERO, ClinicalTrials.gov and WHO ICTRP (searched 5 August 2026; Section 2.3). The effect of the eligibility amendments on study selection was assessed in a post hoc sensitivity analysis reported in Section 3.1 and Supplementary Table S7. The review was not registered, so these are amendments to the internal a priori protocol rather than to registered information.
**Support**	25	Describe sources of financial or non-financial support for the review, and the role of the funders or sponsors in the review.	Funding statement (back matter): National Research Foundation of Korea (2021R1A6A1A03038996) and Gachon University research fund 2025 (GCU-202503210001). The funders had no role in the review.

Section and Topic	Item #	Checklist item	Location where item is reported
**Competing interests**	26	Declare any competing interests of review authors.	Conflicts of Interest statement (back matter): the authors declare no conflicts of interest.
**Availability of data, code and other materials**	27	Report which of the following are publicly available and where they can be found: template data collection forms; data extracted from included studies; data used for all analyses; analytic code; any other materials used in the review.	Data Availability Statement (back matter) and Supplementary Tables S1–S9: all data extracted from the included studies are provided (S1 study characteristics; S2 NOS-CS domain-level quality; S3 full search strategies for every source; S4 AMSTAR 2 appraisal of the two contextual meta-analyses; S5 per-outcome pooling feasibility; S6 citation matrix quantifying overlap; S7 sensitivity analysis of eligibility under the original criteria; S8 analytical parameters bearing on cross-study commensurability; S9 cell-by-cell attribution of the direction assignments in Table 1). No new primary data and no custom analytic code were generated.

From: Page MJ, McKenzie JE, Bossuyt PM, Boutron I, Hoffmann TC, Mulrow CD, et al. The PRISMA 2020 statement: an updated guideline for reporting systematic reviews. BMJ 2021;372:n71. doi: 10.1136/bmj.n71. This work is licensed under CC BY 4.0. To view a copy of this license, visit https://creativecommons.org/licenses/by/4.0/

In addition, three records that had passed full-text assessment were excluded at re-screening against the a priori PICO criteria: one lacked a healthy-control comparator and two did not report an extractable microstate parameter. These are reported by reason and count in Section 3.1 and in the footnote to Supplementary Table S1. Individual citations are not given for these or for the remaining category-level exclusions.
